# Empirical and philosophical problems with the subspecies rank

**DOI:** 10.1002/ece3.9069

**Published:** 2022-07-10

**Authors:** Frank T. Burbrink, Brian I. Crother, Christopher M. Murray, Brian Tilston Smith, Sara Ruane, Edward A. Myers, Robert Alexander Pyron

**Affiliations:** ^1^ Department of Herpetology American Museum of Natural History New York New York USA; ^2^ Department of Biological Sciences Southeastern Louisiana University Hammond Louisiana USA; ^3^ Department of Ornithology American Museum of Natural History New York New York USA; ^4^ Life Sciences Section, Negaunee Integrative Research Center Field Museum of Natural History Chicago Illinois USA; ^5^ Department of Biological Sciences Clemson University Clemson South Carolina USA; ^6^ Department of Vertebrate Zoology Smithsonian Institution, National Museum of Natural History Washington District of Columbia USA; ^7^ Department of Biological Sciences The George Washington University Washington District of Columbia USA

**Keywords:** gene flow, genomics, ontology, reproductive isolation, species, subspecies

## Abstract

Species‐level taxonomy derives from empirical sources (data and techniques) that assess the existence of spatiotemporal evolutionary lineages via various species “concepts.” These concepts determine if observed lineages are independent given a particular methodology and ontology, which relates the metaphysical species concept to what “kind” of thing a species is in reality. Often, species concepts fail to link epistemology back to ontology. This lack of coherence is in part responsible for the persistence of the subspecies rank, which in modern usage often functions as a placeholder between the evolutionary events of divergence or collapse of incipient species. Thus, prospective events like lineages merging or diverging require information from unknowable future information. This is also conditioned on evidence that the lineage already has a detectably distinct evolutionary history. Ranking these lineages as subspecies can seem attractive given that many lineages do not exhibit intrinsic reproductive isolation. We argue that using subspecies is indefensible on philosophical and empirical grounds. Ontologically, the rank of subspecies is either identical to that of species or undefined in the context of evolutionary lineages representing spatiotemporally defined individuals. Some species concepts more inclined to consider subspecies, like the Biological Species Concept, are disconnected from evolutionary ontology and do not consider genealogy. Even if ontology is ignored, methods addressing reproductive isolation are often indirect and fail to capture the range of scenarios linking gene flow to species identity over space and time. The use of subspecies and reliance on reproductive isolation as a basis for an operational species concept can also conflict with ethical issues governing the protection of species. We provide a way forward for recognizing and naming species that links theoretical and operational species concepts regardless of the magnitude of reproductive isolation.

## Graphical abstract

The use of subspecies taxonomy has a long and problematic history in systematics and recent studies using genomic data have suggested their continued use when incompletely reproductively isolated lineages are discovered. We demonstrate that where subspecies represent ontological individuals, they are actually species, and where they are not ontological individuals they do not represent taxonomic units in evolutionary biology. We demonstrate a way forward for understanding lack of reproductive isolation among lineages in a taxonomic context.

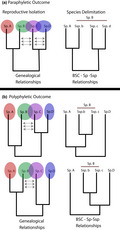

## 1 INTRODUCTION: GENE FLOW AND THE SPECIES PROBLEM

It is now understood that the history of life on Earth is not easily represented as a bifurcating process (Mallet et al., 2016; Wen et al., 2016) and that many organisms fail to maintain genomic exclusivity with closely related or even long extinct relatives (Reich, 2018). Extreme examples of nonbifurcating histories have shown that some species or even entire clades may have been produced from reticulating ancestral taxa over millions of previous generations (Abbott & Rieseberg, 2012; Baack & Rieseberg, 2007; Frantz et al., 2013). Incomplete reproductive isolation provides a biologically interesting landscape of possibilities for speciation, such as adaptive introgression (Figueiró et al., 2017; Leroy et al., 2020; Schmickl et al., 2017) or spatially dependent genetic incompatibilities changing over environments (Barnard‐Kubow & Galloway, 2017). Absence of reproductive isolation after speciation, reflected as continued introgression across parts of the genome, is now well established (Wang et al., 2019; Wu, 2001). While degree of reproductive isolation may increase with time since divergence (Dufresnes et al., 2021), the spatial nature of isolation and the portion of the genome involved in speciation vary widely. Gene flow therefore makes the boundaries between many species indistinct or “fuzzy.” When researchers categorize individuals into taxonomically coherent species, this uncertainty is likely to present difficulty.

The “gray zone” of speciation (de Queiroz, 1998) highlights the broad set of empirical outcomes where sometimes uncomfortable taxonomic decisions must be made or are alternatively ignored altogether. In the gray zone of “incomplete” genealogical exclusivity, uniquely identified lineages may remain connected by occasional or ongoing introgression, making determination of species status difficult when relying on overall measures of gene flow to delimit species (Jackson et al., 2017; Leaché et al., 2019; Nosil, 2008; Roux et al., 2016). Degree of gene flow might be negatively correlated with age of divergence, which on the surface could help identify where lineages are in the gray zone. However, a correlation between time and gene flow may be disconnected by divergent selection at loci due to sexual and ecological pressure (Gavrilets, 2004; Nosil, 2012; Singhal & Moritz, 2013). Additionally it may never be clear when species reach one side of the gray zone (complete collapse) or the other (complete speciation).

In some groups, degree of reproductive isolation scales with time of divergence (Bolnick & Near, 2005; Singhal & Moritz, 2013), but not in others (Burbrink et al., 2021). Pre‐ and postzygotic isolation may also accumulate at different rates (Stelkens et al., 2010; Uy et al., 2018). However, almost 70% of sister species in vertebrates are presently allopatric (Pigot & Tobias, 2015) and the degree of reproductive isolation cannot be tested (Barrowclough et al., 2016). With many taxa existing over 100,000 generations with continuous or intermittent connection between lineages, one should ask: how have these lineages retained their identity for so many generations in the face of gene flow if they are not distinct evolutionary entities (i.e., species)? This is contrasted against known rates of species reversal or extinction by hybridization, which can occur in just a few generations for range‐limited taxa such as various fish groups and Darwin's finches (Hendry et al., 2006; Rudman & Schluter, 2016; Seehausen, 2006; Seehausen et al., 1997; Taylor & Larson, 2019; Vonlanthen et al., 2012) to thousands of generations for species with continental ranges like ravens (Kearns et al., 2018). For other taxa, partial reproductive isolation may be a stable evolutionary endpoint and indicate why species showing ancient divergences with gene flow fail to collapse (Servedio & Hermisson, 2020). In the gray zone of speciation, there are thus crucial questions about how taxonomists should address naming geographic lineages showing spatial overlap and introgression given the complexities of demography, selection, and hybridization (Jackson et al., 2017; Leaché et al., 2019; Roux et al., 2016).

The indefinite nature of many species boundaries has long been recognized (Darwin, 1859; Hey, 2001; Hull, 1976; O'Hara, 1993). To resolve this taxonomic conundrum, many researchers in the 20th century (particularly during and immediately after the Modern Synthesis) inferred reproductive isolation and applied the rank of subspecies to diagnose, define, and delimit populations with fuzzy boundaries (Mayr, 1965, 1982). The use of subspecies to represent geographic variation has a long history in systematics from the late 19th century through to the present. As early as the 1950s, however, problems with the subspecies solution had been identified (Cracraft, 1983; Frost & Kluge, 1994; Gillham, 1956; Rosen, 1979; Wilson & Brown, 1953).

From a perspective where subspecies are considered entities and not artificial constructs (Cracraft, 1983), the rank might represent something of interest to evolutionary biologists or, alternatively, attempt to preserve the identity of distinct lineages. The latter underscores that those lineages are not fully reproductively isolated and therefore not appropriate for taxonomic recognition as species. Subspecies then represent a placeholder category, expecting either those historical lineages will cease to be unique (collapse) or will eventually become species (incipient species), but without differentiating between these contrasting scenarios in the present day. Both situations implicitly rely on speculation rather than evidence regarding the future trajectory of reproductive isolation (O'Hara, 1993; Zink & McKitrick, 1995). As we explain below, neither view of prospective subspecies taxonomy serves to identify lineages properly or reveal future processes of divergence. We therefore provide a description of what species are, what subspecies are not, and why the lure of the subspecies rank should be resisted if we are to move forward with clear taxonomies that better describe the reticulated tree of life.

As we outline below, synthesizing decades of thought on the philosophical and practical literature of the “species problem,” species are historical entities that are phylogenetically diagnosable and exist as ontological individuals, occupying a unique position in the process of evolution. As such, they are not required by any modern understanding of evolutionary theory to be reproductively isolated as ontological individuals will exhibit leaky or fuzzy boundaries across both space and time. Therefore, replacing species rank with subspecies in cases where the former fail to show reproductive isolation is unwarranted. We therefore assert that the following are indefensible: (1) philosophically, to accept the existence of subspecies as ontologically distinct entities within species; (2) biologically, to recognize subspecies as arbitrary divisions of clines when such units lack an evolutionary basis and phylogenetic diagnosis; and (3) operationally, to use the subspecies category as a pragmatic tool to advance aims such as field guide identifications or conservation policy and management.

## 2 BRIEF HISTORY OF SUBSPECIES AND REPRODUCTIVE ISOLATION

The rank of subspecies has a long history of discussion and implementation under fundamentally different concepts. These concepts range from those without explicit evolutionary interpretation to those being essentially the same as species. Subspecies represented as trinomials have been applied at least since 1844 (Remsen, 2010; Simpson, 1961) and were considered to be essentialistic, similar to the rank of species at that time (Mayr, 1982). After Darwin (1859), subspecies were often considered as natural entities and not classes. Subspecies were thought to be incipient species by some authors (Rensch, 1928, 1929; Rothschild & Jordan, 1895, 1903) which are part of species, or *Rassenkreis* (circle of races; polytypic species or differences at the ends of isolation by distance; Reydon & Kunz, 2021). Early workers such as Gloger, Bergmann, and Allen viewed subspecies as adaptive geographic variants to be applied when addressing ecogeographic phenomena (see reviews in Mallet, 2013; Mayr, 1982). Subspecies descriptions increased throughout the late 19th to mid‐20th century biased toward European and North American mammals, birds, butterflies, and to a lesser degree reptiles and amphibians (Burt, 1954; Frost, 2020; Frost & Hillis, 1990; Gillham, 1956; Mayr, 1946; Padial & De la Riva, 2021). This taxonomic bias not only is notable given the small contribution of these groups to the overall biodiversity of life on Earth, but also expected given the emphasis on studying these organisms in the Northern Hemisphere (Mora et al., 2011).

In many cases, previously diagnosed morphological species named by earlier researchers were demoted to subspecies and considered geographic variants of widespread species (Stresemann, 1975). Proliferation of subspecies names continued through the middle 20th century, when arbitrary sections of clines and minute phenotypic variants were formally named in many groups (Burt, 1954; Gillham, 1956; Huxley, 1938; Padial & De la Riva, 2021). For example, in reptiles, subspecies were described at their highest rate after the 1950s and declined rapidly toward the end of the 20th century (Uetz & Stylianou, 2018). This is paralleled in ornithology, where subspecies descriptions increased from the late 19th century, peaked in the mid‐20th century, and declined rapidly toward the 21st century (Remsen, 2010).

Wilson and Brown (1953) struck back at the widespread proliferation of subspecies by showing that (1) they are often defined by an arbitrary choice of characters that can differ widely over geographic space, (2) the same characters often occur in different areas of a species' range, (3) microgeographic races are a common outcome of elaborate and extensive trait variation due to local adaptation, and (4) there is a lack of a lower limit for defining these entities. Essentially, any number of arbitrary traits can be used to group individuals into an arbitrary number of subspecies. However, champions of the subspecies idea continued (Mayr, 1954; Parkes, 1982; Smith & White, 1956). In fact, the years immediately following Wilson and Brown (1953) and Brown and Wilson (1954) saw a “cline” of opinions from authors wanting to eliminate the rank to those wanting to produce more refined definitions. Some authors considered only established allopatric forms as subspecies, whereas others devised rules to handle arbitrary descriptions (Burt, 1954; Edwards, 1954; Gosline, 1954; Inger, 1961; Starrett, 1958).

The taxonomic rank of subspecies has been defined and redefined for many decades (Amadon, 1949; Braby et al., 2012; Mayr, 1965; Patten, 2015; Rand & Traylor, 1950), though there has been little consistency in the criteria used to delimit subspecies boundaries. Various rules have been proposed to delimit subspecies believed to be more meaningful than arbitrary handles of convenience. Some authors consider subspecies to not be evolutionary lineages, equivalent to evolutionary lineages, former evolutionary lineages, or rank‐free evolutionary lineages (Amadon, 1949; Braby et al., 2012; de Queiroz, 2020; Hillis, 2020; Mayr, 1965; O'Brien & Mayr, 1991; Rand & Traylor, 1950). Often they are simply recognized as unique para‐ or peripatric subdivisions within the range of a species defined by phenotypic similarities that are composed of fertile individuals. Other authors only consider allopatric populations as candidate subspecies (Edwards, 1954; Haig et al., 2006). A recent review of the many ways subspecies rank is defined suggests that they show ecological, morphological, or genetic trait differences often over geographic space with some degree of reproductive (in)compatibility (Reydon & Kunz, 2021).

Several methodological approaches using morphological, ecological, or genetic data to decide when lineages should be delimited as subspecies have been applied over the last 70 years. For example, Amadon (1949), Mayr (1969), and Patten and Unitt (2002) proposed a threshold where 75% or more of individuals examined in one population lie outside the 99% range of another population. O'Brien and Mayr (1991) recommended that subspecies not only be allopatric and receive no migrants, but also possess exclusive phenotypic characters defining a unique natural history. Other definitions regard subspecies as distinct populations with at least one phenotypic trait diagnosable in at least 95% of individuals (Remsen, 2010). Tobias et al. (2010) used a phenotypic yardstick when measuring morphological and vocal traits in birds to generate a minimum threshold for sympatric and parapatric species. Köhler (2021) advocated combining mtDNA phylogenetic tree structure with sequence divergence thresholds to delimit species versus subspecies, though no criteria are given for the spatial distribution of taxa or degree of reproductive isolation. Rather, taxa are ranked in a tree and then genetic divergences are assessed over various ranges of values thought to represent species or subspecies. Others have suggested that subspecies be allopatric, divergent along at least one axis of genetic, morphological, or ecological variation, but “less” than what would be expected for closely related species existing in sympatry (Descimon & Mallet, 2009). Additionally, subspecies have been conceived to reflect a range of incomplete adaptive divergence within species that do not rise to the “level” of specific differentiation Braby et al., 2012).

Others have recently tried to establish the link between phenotypic and genomic differentiation of populations when identifying subspecies (Patten, 2015). These “subspecies genes” (the term used by Patten, 2015) are considered discoverable using genomic methods. In parapatric populations, “subspecies genes” are thought to provide evidence that these entities represent incipient species. For parapatric subspecies, allelic introgression is expected to vary widely with neutral alleles moving extensively between populations and adaptive alleles remaining local to each subspecies (Braby et al., 2012). Most recently, Dufresnes et al. (2021) suggested that the distribution of cline widths among diagnostic SNPs be used to determine if lineages represent species or subspecies. Here, Poisson or binomially distributed densities abutting widths of 0 km indicate the presence of two unique species with genes likely tied to reproductive isolation, whereas Gaussian‐distributed densities centered on larger widths are indicative of subspecies.

It is clear that most modern proposals identifying subspecies as being different from species rely on perceived lack of reproductive isolation (Braby et al., 2012; Mayr, 1965, 1982). However, most described species have never directly been tested for degree of reproductive isolation in any meaningful way (Cracraft, 1983; Mayr, 1963), and most sister species of vertebrates are allopatric and therefore cannot be tested (Pigot & Tobias, 2015; Zink, 2014). For example, avian taxonomic classification committees for North and South American birds, which follow the Biological Species Concept (BSC), use a range of criteria to delimit species. A review of how bird species were delimited in practice found that diagnosability was the most frequently applied criterion (Sangster, 2014). As Mayr (1963) points out, the application of the typical morphological species concept (species differ enough morphologically to be considered unique) is simply serving “as secondary indications of reproductive isolation.” It follows then that this view of morphological, behavioral, and molecular differentiation are often surrogates for identifying reproductive isolation when being applied to determining subspecies rank. Therefore, most instances of species and secondarily subspecies description fail to directly test for reproductive isolation, but rather infer it given degree of difference in measured characters.

Because reproductive isolation is usually not tested does not mean that such testing is impossible given behavioral and genomic data and modern computational methods (Turbek et al., 2021). Reproductive isolation is fascinating as a biological process, even though it is not in of itself a “trait” possessed by any species (Coyne & Orr, 2004), but rather as a measure of interaction as a result of speciation. However, studying reproductive isolation necessarily requires the presence of two entities. This underscores the obvious point that historical lineages have to be defined independently of reproductive isolation to be able to quantify the supposed lack of independence (Cracraft, 1983; Nelson & Platnick, 1981). Identifying these independent lineages is a necessary first step before quantifying hybridization over a landscape.

Failure for reproductive isolation to occur between lineages continuously distributed over the landscape often results in some form of a hybrid zone. These zones can be examined to understand if reproductive isolation is actually occurring given the observed hybridization. Thus, if endogenous or exogenous selection is present, then species boundaries are likely to be preserved. Realistically, the degree of reproductive isolation, extent of linkage disequilibrium, and amount of backcrossing is not easily determined given that hybrid zones change widths, extent, and location through time (Ryan et al., 2018). Reproduction through a hybrid zone could reflect true neutrality where species might collapse, be reinforced in the case of selection against hybrids (tension zones), or reveal gradients of environmental selection from one parental species to the other parental species (Barton, 1979; Barton & Gale, 1993; Endler, 1977; Gompert et al., 2017; Harrison & Larson, 2014; Nachman & Payseur, 2012). Moreover, hybrid zone widths alone may not be reflective of the degree of reproductive isolation because the sizes and location of the zone may change over several orders of magnitude considering variation in dispersal rates, historical climate change, and positioning of density troughs between species that attract hybrid zones (Barton & Hewitt, 1985; McEntee et al., 2020). Therefore, there may be no clear pattern suggestive of lineage collapse or complete reproductive isolation indicated by these studies.

Changes in hybrid zone shapes and locations over time might be common (Buggs, 2007; Ryan et al., 2018; Wielstra, 2019), as revealed by evidence from the fossil and pollen records, niche modeling through time, displacement of extant populations of one species from the expanding range of another, or genome‐wide evidence from displaced lineages. There is ample evidence that hybrid zones of various shapes and sizes have existed from the present through to the late Miocene between extant species (Barth et al., 2020; Burbrink et al., 2021; Hewitt, 2011). In birds, fertile hybrids can be produced well past speciation, even among taxa sharing a common ancestor more than 17 million years ago (Prager & Wilson, 1975; Price & Bouvier, 2002). Importantly, evidence from the predicted origin of hybrid zones along with continuous or repeated instances of contact suggests that hybrid zones have formed and reformed many times, yet the identity of the interacting lineages remain intact despite gene flow (Wang et al., 2019). As pointed out by Servedio and Hermisson (2020), partial reproductive isolation may be a long‐term stable reality for most species. Gene flow may never reach a point of species collapse or absolute reproductive isolation, therefore rendering the subspecies category again superfluous when unique evolutionary histories of species are maintained over millennia. This is in contrast to documented species collapse that occurs just in a few generations upon secondary contact (Rudman & Schluter, 2016; Seehausen et al., 1997; Taylor et al., 2006; Vonlanthen et al., 2012).

Complete reproductive isolation is not the universal indicator of speciation, nor is it necessary or even common for “good” species that form and maintain their evolutionary distinctiveness over time. Defining what is meant by reproductive isolation is often complex given differential introgression throughout the genome and unique interactions over time and space along hybrid zones. At least for methods described earlier that use some quantification of hybridization or gene flow, only arbitrary breaks along a continuum of reproductive compatibility can “determine” when evolutionary lineages represent subspecies or species (Dufresnes et al., 2021; Hillis, 2020; Tobias et al., 2010). Unfortunately, none of these proposals have considered how subspecies and species actually differ with regard to ontology or process (Burbrink & Ruane, 2021; de Queiroz, 2022).

## 3 SPECIES ONTOLOGY AND ITS CONSEQUENCES FOR SUBSPECIES

### 3.1 Are species ontological individuals?

Debates over taxonomy and the species problem are inherently philosophical; an infinite amount of data and methods (“epistemology;” ways of knowing) can never answer these basic questions (see Hull, 1990). Here, we provide a brief introduction of history and background of philosophical inquiry into these issues. However, the concepts herein are crucial for an understanding the nature of species. Chief among these is “ontology”; the nature of being and what “kind” of things exist and what can be known. Only after the ontology of species is circumscribed (ideally from an evolutionary basis) can we ask the epistemological question: how do we define, delimit, describe, and diagnose them?

Because the subspecies rank is inherently tied to the species problem, we compare the ontology of species and subspecies regarding how we detect, diagnose, delimit, and define them given various species concepts. We hold that species are natural concrete objects and are not abstractions (Ghiselin, 1974, 1997; Hey, 2001; Nathan & Cracraft, 2020). That is, they are real entities that exist in the real world. Species are fundamental units of evolution that are also the fundamental rank in the taxonomic hierarchy (Bock, 2004). de Queiroz, (1997) noted that this special status decouples species from the hierarchy of taxonomic ranks. Therefore, this rank occupied by species in the otherwise‐arbitrary hierarchy of taxonomy coincides with a biologically meaningful unit, unlike other ranks such as genus and family. Thus, species are real and are the aim of discovery of taxonomy, while the remaining higher ranks are applied to named clades of increasing inclusiveness as an approximation of their evolutionary history (Hennig, 1966). However, if species are parts of clades at different levels of inclusiveness, and these clades are also considered as individuals, then assigning species to higher named taxa is not classification, in the sense of class versus individual (de Queiroz, 1988, 2005).

A key concept here is the argument that species are ontological “individuals”; discrete or separate objects which may be similar to others but nevertheless have independent and unique identities. You, the reader, are an individual person among several billion. Similarly, the species *Homo sapiens* is an evolutionary lineage representing one among several in the family Hominidae. The individual identity of *H. sapiens* has a similar nature to your own. In contrast, the idea of a class is a set of objects that do not share a *fundamental* identity, but only belong together based on shared attributes. In this view, *H. sapiens* would merely be any bipedal mammal with speech, culture, 23 pairs of chromosomes, etc., of which you happen to be an instance, but does not have any more inclusive or meaningful nature or historical identity. Such a view is, we (and others) suggest, incompatible with the existence of species as the outcome of evolutionary processes.

The recognition of species as ontological individuals has a long history (Baum, 1998; Bernier, 1984; Brogaard, 2004; Coleman & Wiley, 2001; Ereshefsky, 1992; Frost & Kluge, 1994; Ghiselin, 1974, 1981, 1987; Hennig, 1966; Holsinger, 1984; Hull, 1976; Kitcher, 1984; Mayden, 2002; Mishler & Brandon, 1987; Queiroz, 1999; Rieppel, 2009; Rieppel & Grande, 2007; Wiley, 1980). The implications of individuation versus the treatment of species as classes/natural kinds have been detailed elsewhere (Frost & Kluge, 1994; Mayden, 2002). To review, if species are ontological individuals, they must fit specific criteria for the category. We consider the criteria for individuation to be the following: Is it ostensively defined? Is the thing a particular? Are there instances of the thing? Is it bounded in space and time, with the boundaries being fuzzy? Do the parts exhibit cohesion? Is the thing a mereological sum (Table 1)?

**TABLE 1:** Criteria that differentiate ontological categories of individual and class (also see text)

	Example
Individual	
Particular thing	*Lithobates heckscheri* – The river frog represents a unique entity
No instances	One lineage of *L. heckscheri*
Defined through ostension	Can point to unique diagnostic characters
Bound in space and time	Distributed only in SE North America, diverged from closest living relative ~15–10 mya
Cohesive	Individuals of *L. heckscheri* are connected via tokogenetic processes; changing adaptive landscapes affect all individuals
Mereological sums	Composed of other individuals; individual organisms of *L. heckscheri* are parts of the whole lineage
Class	
Universal thing	Hydrogen (H) atom – a kind or type of object, not unique
Instances exist	H atoms are exactly the same, and can be created
Defined through intension	H defined by strict rules, but not by a fundamental identity
Not spatiotemporally bound	H originated with the universe, found across universe
Not cohesive	Single H affected at a time; nothing affects “hydrogen” as a whole
Not mereological sums	Not parts of wholes, the parts of H are also class objects

Species are particular things, so there are no instances of them. They are not universals like “chairs,” of which there are many instances. The River Frog *Lithobates heckscheri* is a unique thing, a particular of which there are no instances. Species are not defined by a specific list of characteristics or rules that will always *define* a species, that is, they are not intentionally defined. Contrast that with hydrogen, which is always defined by the presence of a single proton and a single electron. Species have diagnostic features that allow us to *point to* and say “that is *Lithobates heckscheri*.” As such, species are ostensively defined by reference to individuals, and are therefore *diagnosed* rather than being classified or characterized by the recognition of intensionally defined attributes possessed by their members. Species are spatiotemporally bound, they have beginnings (speciation) and ends (extinction). The boundaries in space and time are fuzzy. The fuzziness refers to geographic distribution and tokogenetic reticulation (migration of individual organisms) between lineages. Consider hydrogen again, which likely appeared at the beginning of the universe and continues to exist throughout the space of the universe. The parts of species exhibit cohesion through the tokogenetic nexus and respond to similar processes in similar ways.

If species are individuals and their parts are also individuals, then species are mereological sums, which refers to part–whole relations; are species more than the sum of their parts – the living members? Each organism within a species is a particular thing, an ontological individual. If each organism is a part of a species, then species would be a whole ontological individual composed of its parts, the specific organisms as ontological individuals. Based on the criteria for arguing that a thing fits the ontological category individual, species are individuals.

### 3.2 Are subspecies real and individual?

A way to answer this is to ask if subspecies exist without human perception. Evolutionary lineages are things that existed prior to and independently of us observing them. Lacking perfect knowledge of evolutionary history, we are left to interpret phylogeny, taxonomy, and species delimitation by observing limited data such as DNA and phenotype. For a subspecies that is delimited using such data, we can then ask how we would interpret this given perfect knowledge of evolutionary history and genealogy. If such knowledge revealed that the subspecies was in fact a unique and independent lineage, it would then rightfully be considered a species. If instead we learned that a subspecies was a class of individuals exhibiting characteristics such as a phenotype due to contingent circumstances (e.g., local adaptations), these would not represent ontological individuals and would not merit taxonomic recognition.

There is another level to the reality of subspecies, having to do with the name. We see a dissonance between subspecies as trinomials and the biological entities they have been purported to be. Take *Agkistrodon contortrix contortrix*, which is a real name, just as Hamlet and Clarissa Dalloway are real names. However, the things they represent are not real. If *A. c. contortrix* is a distinct, concrete entity in nature, an independent evolutionary lineage representing an ontological individual, then it is simply *A. contortrix*, a species. If subspecies are considered a kind of evolutionary unit, the recognition of subspecies as a class would reject that claim because evolution as a process would not exist for subspecies: no evolutionary processes, then no evolutionary unit. If *A. c. contortrix* is a class of *A. contortrix* specimens showing a particular color pattern (*sensu* Gloyd & Conant, 1990), then *A. c. contortrix* is not a real entity in nature and is just asregardless if unrooted fears of undefined “fictional as the Prince of Denmark and the nostalgic hostess from Westminster. We are left with the conclusion that if subspecies are indeed real things and individuals, then they are species.

Therefore, we take a skeptical approach to the notion that subspecies exist but are *not* ontological individuals. We use the specific criteria for the category ontological individual (as noted earlier for species) to challenge the idea that subspecies cannot be individuated. So the questions below, directly and one criterion at a time, evaluate subspecies as individuals.

Are subspecies ostensively defined? This question stands out for subspecies because the way many workers name subspecies is based on some theoretically localized morphological variation (that species is blue over there, but not here), thus you can point to the blue feature and name it. Given that a subspecies can be diagnosed in this way, it is actually a species; the subspecies rank does not stand as a distinct and separate real, concrete individual aside from the species. It is important to note that we do not think that species are the only biological individuals. Monophyletic groups are diagnosable and are biological individuals. Subspecies, however, are not monophyletic and again, as such, not diagnosable.

Are subspecies particular things without instances? There are two ways to address this. Subspecies could be instances of species, but if species have instances then species must be classes. However, species are not classes, they are individuals, and do not have instances. If subspecies are defined by specific rules, say the presence of blue members, and demes or populations of blue members that exist in disconnected space, then subspecies would be a class with instances of each other. If subspecies are unique evolutionary units, and thus particulars, then subspecies would be an individual and a part of a whole. That means they would also be diagnosed and not defined by a set of rules. As such, subspecies would again be indistinguishable from species.

Are subspecies spatiotemporally bound, with the boundaries fuzzy? If subspecies are evolving units such as incipient species or as lineages collapsing via hybridization, then they certainly would be bound in time and space with fuzzy boundaries. Incipient species and collapsing lineages reflect lineage dynamics as diverging and merging parts of the tokogeny, respectively. We assume which parts of the tokogeny are named as subspecies based on reproductive connectivity, but where do these subspecies begin and end? And how do these processes differ from the process of lineage reticulation? We are left to conclude that markers of spatiotemporal boundaries are artificial (i.e., where and when are organisms blue) and in fact simply reflect a normal process inherent to lineages that are species.

**FIGURE 1:**
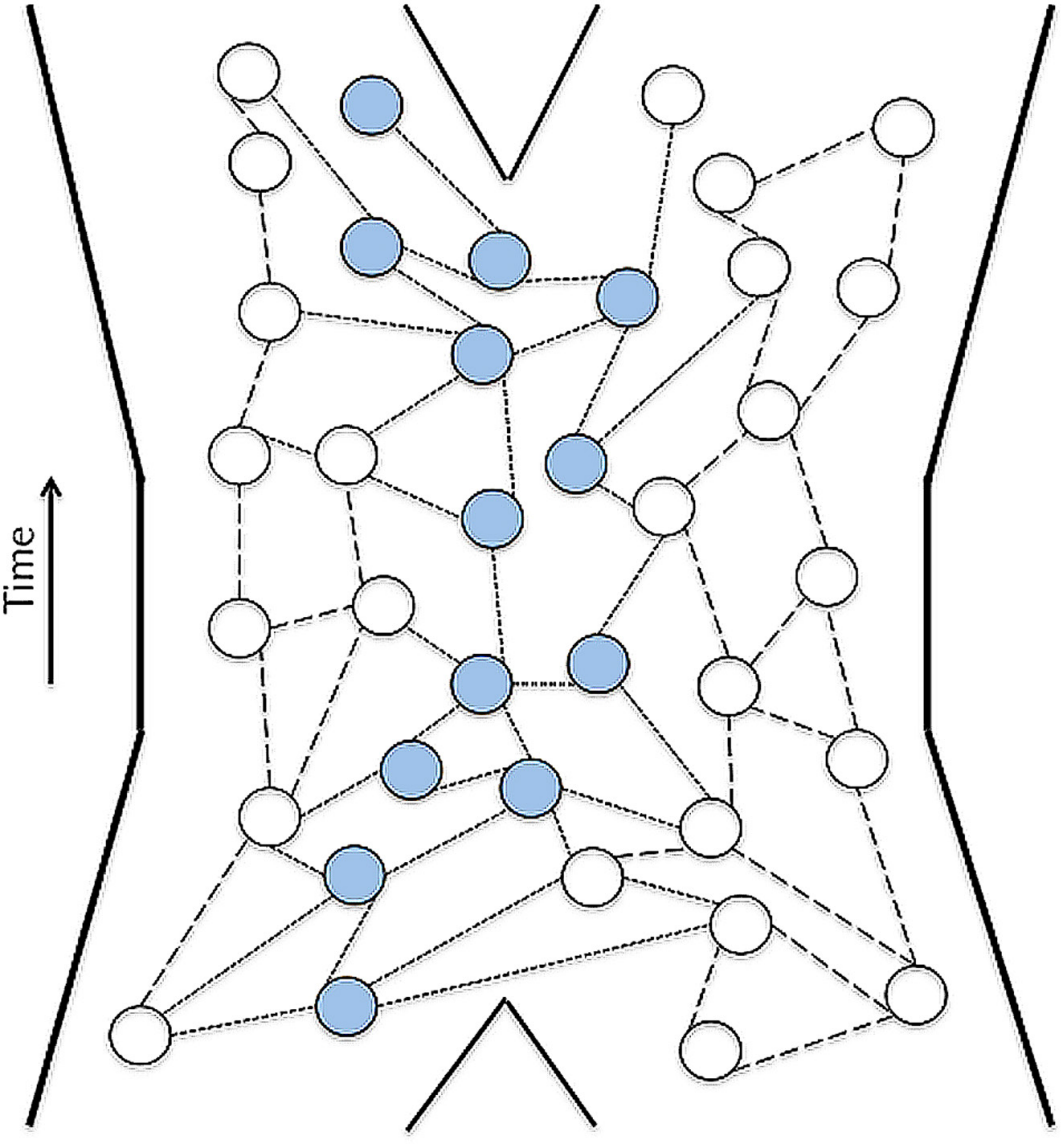
A schematic illustrating the partial cohesion, partial boundedness, and the partial participation as interactors of a subspecies within a lineage. The tokogenetic nexus (dashed lines) depicted contains all circles (organisms) and their replicating connection between them is illustrated through lines. The blue dots depict the delimited individuals through time to be members of a subspecies with which other members of the tokogeny reproduce but are not included (dotted lines), illustrating partial participation within a real ontological individual

Do subspecies exhibit cohesion? We think they must, but only partially, regardless of how they are delineated within a species. If they were fully cohesive, they would be recognized as species. However, in the delineation, other cohesive parts of the whole lineage (the species) are intentionally left out. So, some parts/members of the subspecies may be responding cohesively with extralimital parts, thus rendering the cohesion partial (Figure 1).

Are subspecies mereological sums? Subspecies must be individuals composed of parts which are individuals to be such. Subspecies are certainly composed of ontological individual parts (i.e., each organism). If the subspecies does not have instances and is spatiotemporally bound, then such an entity composed of these parts would be a mereological sum. Would that entity still be a subspecies? No, that entity would be a species.

Specifically, for those subspecies that are allopatric historical lineages, these are no different from species (see Collins, 1991). For those subspecies considered historical lineages as either incipient or merging species, they, too, are ontologically no different than species evolving as part of the phylogeny. We note that assessing these processes with data under any concept (e.g., BSC, Evolutionary Species Concept, and Phylogenetic Species Concept) implicitly contain prospective statements (O'Hara, 1993). For example, a group of populations that qualify as a species in the present moment is predicted to continue instantaneously into the immediate future. Even if they begin to merge over time and eventually cease to be distinct species, this will not happen instantaneously, as they are spatiotemporally distinct. Even at nearly instant temporal scales, interpreting subspecies as incipient species already suggests that spatiotemporally independent lineages are cohesive and thus species. Therefore, subspecies as former historical lineages that are in the process of merging are also species.

### 3.3 Can there be a subspecies concept?

Considering species as the fundamental units of evolution that are also concrete individuals, we then ask if they can be discovered under a single or multiple concepts. The idea of monism suggests species are discoverable by one concept (Hull, 1999). This is in opposition to pluralism, where a single concept cannot account for various processes that generate species in different groups. Monism aligns well with concretism and suggests that among the plethora of species concepts used today, these generally represent different practical instantiations of a single underlying concept that are theoretically and operationally practical for defining species (see de Queiroz, 2007; Nathan & Cracraft, 2020). Alternatively, perhaps the actual “species concept” has not yet been discovered. Reydon (2006, 2005) suggested that a pluralistic view of species may be at the heart of debates about the species problem. Under this pluralistic view, species may be considered as four different kinds of entities: (1) synchronic – equivalent to biological species, (2) diachronic – segments of the tree of life, equivalent to phylogenetic species, (3) classes sharing similar properties, or (4) classes of evolving populations or groups. Here, the first two categories are considered individuals and may not actually be different kinds of entities, but rather viewed as time limited or time extended (de Queiroz, 1988, 1998). The second two are classes. Within diachronic species, there exist two other categories differentiating between lineages and clades. The former are lineages that are reproductively compatible, and Reydon and Kunz (2021) treated both lineages and clades as biologically relevant. Subspecies would be diachronic and also equivalent to species in that regard.

Considering species as evolving individuals should be recognized as the dominant and necessary basis for evolutionary classification. However, the BSC continues to cast a long shadow over species delimitation, though instances where the criterion of reproductive isolation is actually rigorously tested empirically when delimiting species are rare (Cracraft, 1983). For the most part, phenotypic differences served to indirectly determine if species were potentially interbreeding (Sokal & Crovello, 1970) until the rise of genetic data. The vast majority of named species are likely also distinct evolutionary entities, as taxa delimited based on apparent reproductive isolation are probably separate species in most instances. Of course, these species may also contain multiple independently evolving lineages – cryptic species.

In contrast, this operational basis for classification (reproductive isolation) is also associated with the use of “subspecies” for numerous lineages in the gray zone of speciation, a trend that is still being advocated in several major groups of organisms (Braby et al., 2012; Hillis, 2019; Patten, 2015). However, as noted here and by previous researchers (Cracraft, 1983; Frost et al., 1992), prioritizing a particular form of cohesion over evolutionary history represents a major starting point for problems with recognizing species (Velasco, 2008) and, in particular, promotion of the subspecies rank.

From a classification point of view, where members of a particular class are defined by essential properties, lineages connected by some amount of gene flow could be problematic. But ontologically, species are not classes under most recent interpretations. Species represent the basal category of taxonomy, yet are defined ontologically as individuals (de Queiroz, 1988; Griffiths, 1974). Further subdividing this category has no meaning given that anything below this category is not defined as an individual or simply refers to arbitrary additional gradations of individuals. Logically, if one can group populations and those are identified as spatiotemporal individuals that are cohesive with fuzzy boundaries, then this entity cannot be further subdivided into “species.” Along the continuum of “subspecies” definitions, they either represent nothing concrete in nature or they are species. We thus assert that species are a reasonably indivisible unit; not that variation does not occur within species, but that it does not make sense to consider the existence of infraspecific evolutionary units in taxonomy.

Our assertion thus derives from the nature of species as concrete natural objects which are ontological individuals. This illustrates that taxonomy is the process of identifying the singular real, distinct entities in nature produced by evolution, which are named as species. The category of species is not arbitrary, though taxonomic ranks above the species are arbitrary monophyletic groups. Crucially, this implies that there logically cannot be an ontologically meaningful subspecific entity that is recognized taxonomically. If the subspecies is an ostensively defined individual, it is redundant with the species, and is itself a species (de Queiroz, 2020). If the subspecies is an intentionally defined class, then it is describing intrinsically different levels and hierarchies of biological phenomena which taxonomy is explicitly not attempting to address, such as ecology, behavior, and phenotype. Obviously, species can contain geographically structured genetic sublineages, populations, demes, and individuals, all of which vary from one another in biologically meaningful ways. But the aim of taxonomy is to reflect an evolutionary classification beginning with the fundamental unit of evolution, the species. Infraspecific variation, even if biologically meaningful (e.g., local adaptations) are of a qualitatively distinct nature; we cannot identify any potential subspecific entity that is (1) real and (2) not a species.

If these were not true, and taxonomy were used to delimit hierarchical, class‐based infraspecific variation, there would thus be no logical reason to stop at a single rank below the species. There would instead be an explicit need for an infinite number of infraspecific ranks, sub‐subspecies, sub‐sub‐subspecies, etc., down to taxonomic recognition and nomenclatural allocation of individual organisms within species, or even individual cells or alleles within individuals, as each of these represents the potential substrate for future evolutionary variation or distinctiveness. One might also argue for the taxonomic recognition of other nonspecies entities that provide the context for evolution, such as ecological communities, colonial organisms, or multispecies consortia such as biofilms. Rather, we argue that the existence of ontologically meaningful subspecies is logically impossible.

## 4 RECENT PROPOSALS REVIVING SUBSPECIES

Most modern definitions of subspecies, particularly those that consider genetic data, attempt to bridge evolutionary history with reproductive isolation (Braby et al., 2012; Hillis, 2020). Conceptualizing subspecies under a variety of processes that can be modeled and applied to classify evolutionary history can be problematic. Spatially, subspecies can be peripatric, parapatric, or, by some authors, only allopatric. They can also be incipient species, merging historical lineages, or be unrelated to historical processes that generate unique lineages. As various authors have pointed out for over 40 years, these definitions are almost always unsatisfactory (Frost & Kluge, 1994; Rosen, 1979). As noted by ourselves and other previous authors, this creates a “burden of heritage” that retains subspecies as artificial designations in many modern taxonomies (Crifasi, 2007; Pyron & Burbrink, 2009; Torstrom et al., 2014).

Several recent proposals have been written to revive the use of subspecies in systematics. Hillis (2020) suggested that continuously distributed geographic races that represent formerly isolated lineages be considered subspecies. He favors naming those formerly distinct evolutionary lineages that are apparently being subsumed within the species as subspecies, denoting both historical lineage independence and current nonindependence given a lack of reproductive isolation (Hillis, 2019). After lineages collapse into single species, evidence of their existence will become artifacts represented only as ghost lineages in admixtured populations (Ottenburghs, 2020). However, extinct taxa are still named as species regardless of how they become extinct, even if by hybridization. Therefore, there is no reason to not consider these overlapping lineages as species given that they can still currently be detected as spatiotemporal individuals regardless of gene flow. That they can be detected indicates they are unique evolutionary lineages; they are species regardless of what happens in the future. The benefits of naming species now and properly enumerating biodiversity at the correct scale of classification is much greater than the uncertain drawbacks of either collapsing species or waiting for them to become “more” of a species at some time in the future.

A primary objection to the Hillis (2020) proposal is that he treated the existence of real, historical lineages as an empirical epiphenomenon (“subspecies”) that is distinct from their ontological divergence into separate individuals (“species”). Specifically, a subspecies as Hillis proposed operates like a class to which organisms belong, rather than an individual. Indeed, he stated “A third solution is to use the subspecies category to refer to geographic races. Why would we want to do this? Many applications, such as field guides, rely on the appearance of organisms for identification” and “the subspecies category (or common names) can be used effectively to differentiate geographic races within a species whenever that is practical or important.” Consequently, subspecies are at least permitted (if not required) to be classes defined intentionally by the possession of characteristics such as geographic origin, external morphology, or specific allele frequencies. Yet, these classes are nevertheless defined *within* ontological individuals (species). This logical incompatibility is not necessarily fatal, but we suggest it is incongruous when trying to understand the evolutionary process and use taxonomy to express phylogeny. Therefore, Hillis (2020) would suggest applying the rank of subspecies to cases where historical lineages still retain gene flow, whereas we strongly recommend they be recognized as distinct species given our ontological argument and empirical evidence that species clearly retain the historical signal of independence regardless of introgression.

Despite strong advocacy for subspecies from authors such as Braby et al. (2012) and Hillis (2019, 2020), theoretical work that explains how subspecies form and transition into species has been absent. The lack of a theoretical basis for identifying how “subspeciation” and the maintenance of subspecies differs from speciation is evident (but see de Queiroz, 2020). This is in part a consequence of the lack of a consensus view on how to define subspecies and how to delimit them, as described above. By contrast, evolutionary theory on populations and species, the hierarchical scales below and above subspecies, have a rich legacy and remain active areas of research in speciation and macroevolution. Without a theoretical basis, the relevance of subspecies in evolutionary biology is relegated to a taxonomic rank decoupled from process.

de Queiroz (2020, 2021) provided a distinct approach offering viewpoints grounded in the theory of phylogenetic taxonomy. Importantly, he pointed out that there is nothing necessarily that differentiates between ranks; all historical evolutionary lineages are nested within each other. What he therefore argued is that separately evolving meta‐population lineages (species) may themselves contain population‐level lineages (subspecies) that are of the same fundamental kind, all “species.” Therefore, a species may have multiple incompletely separated subspecies that are nevertheless distinct ontological individuals, species within species. This is analogous to a family containing subfamilies; both describe a fundamentally similar level of variation. In a system of phylogenetic nomenclature (de Queiroz, 1997; Laurin, 2008), ranks are not needed, and we can view all of these historical lineages as ontological individuals nested along the phylogeny.

We differ from de Queiroz in discarding the label of “subspecies” primarily due to historical baggage (Pyron & Burbrink, 2009), though we both seem to recognize the same individuals as “species.” What de Queiroz defined as subspecies, we simply take to be the boundary or limit of ontological definitions of species, suggesting that this can fulfill most needs of the term. Where de Queiroz would call an incompletely separated lineage a “subspecies,” we would simply reiterate that there exists a continuum of divergence between species, ranging from weak to strong reproductive isolation. In summary, we believe that there are few significant disagreements between our view and de Queiroz's, other than that we find his continued support of the word “subspecies” to be an unnecessary complication with an excessive burden of heritage.

We note there remains another option which both the Hillis (2020), de Queiroz (2020, 2021) proposals consider but do not address directly. An ontologically complete philosophy could recognize all spatiotemporally discrete population units as species (Kizirian & Donnelly, 2008; D. Kizirian, *pers. comm*.). This status could be gained and lost instantaneously; a newly formed allopatric island population or geographic population isolate would therefore immediately become a species, but also immediately merge back into the ancestral species upon reconnection (Murray & Crother, 2016). Because such proposals have occasionally been considered (e.g., Collins, 1991), they are generally rejected as being empirically unwieldy and causing taxonomic inflation beyond the level with which most researchers are comfortable. In fact, Hillis (2021) criticized de Queiroz (2020) by suggesting that the latter's proposal would result in something akin to this scenario, in which ever‐finer population structure is delimited as species. de Queiroz (2021) denied this, but admitted that his own threshold for demarcating the continuum between “structure” and “subspecies” remains poorly defined. Arguments appealing to what is useful to humans are irrelevant if recognizing the existence of a particular phenomenon is a goal of science regardless if unrooted fears of undefined “taxonomic chaos” ensue (Hillis, 2020; see Sangster, 2009). We note that naming fine‐scale population structure as subspecies is no less “chaotic” in the sense of proliferating additional names with fuzzy definitions.

## 5 SUBSPECIES PRESENT PROBLEMS FOR CLASSIFICATION AND COMPARATIVE METHODS

**FIGURE 2:**
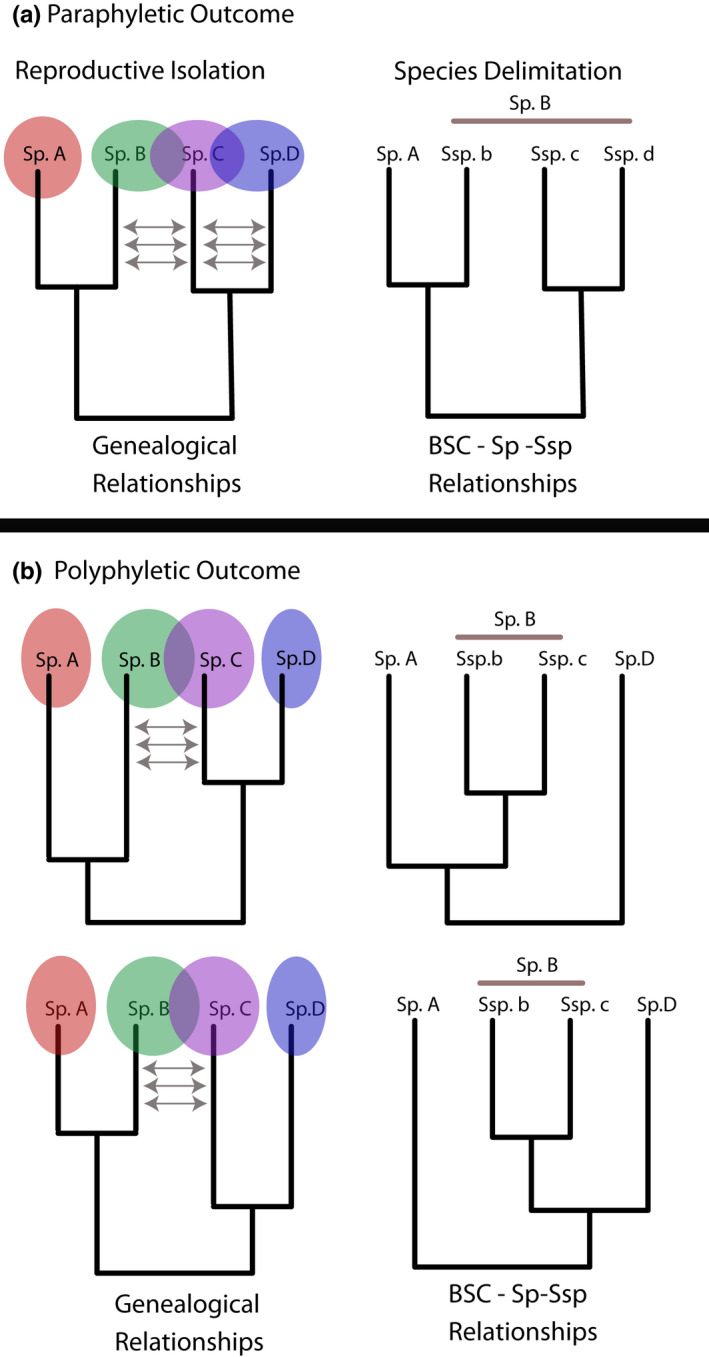
Examples of how recognizing subspecies can distort representations of phylogenetic history. On the left hand side of both panels (a) and (b), the overlap between colored circles indicates lack of reproductive isolation (RI) and is illustrated over the correct genealogical relationships with a thin gray arrow representing hybridization after speciation. (a) A paraphyletic outcome where species are delimited using the biological species concept (BSC) and subspecies are recognized. On the right hand side, the three species (b–d) are considered subspecies of (b) given lack of RI and force a paraphyletic representation of lineages (species and subspecies – *sp*–*ssp* relationships). The sister lineage of species A, subspecies b, is incorrectly constrained to be a lineage within species B. (b) An outcome where species are delimited due to lack of RI and the species, B and C, are constrained to be subspecies of B. Two polyphyletic outcomes are shown where species B is constrained to include two lineages (subspecies b and c) and is either the sister taxon of A or D. However, in either topology species B will contain at least one lineage that is not sister to that species. For example, if species B were considered as sister to species A, then species C can no longer be correctly inferred as the sister lineage to species D

Determining if evolutionarily distinct groups are unique “enough” to merit species status given degree of reproductive isolation disregards historical uniqueness of lineages. In many cases, upholding the primacy of reproductive isolation can distort evolutionary history by applying an incorrect taxonomy to paraphyletic groups (see Figure 2). In instances where biological species group nonsister lineages because of failure to be reproductively isolated, the result is paraphyly (Frost & Kluge, 1994; Rosen, 1979). Application of the subspecies rank to indicate the presence of lineages with gene flow has unfortunately been used to derive paraphyletic classification of the North American Ratsnakes as a valid taxonomic solution (Hillis & Wüster, 2021). Some authors indicate that the concept of paraphyly properly only applies to interspecies relationships (Nixon & Wheeler, 1990; Wiley, 1981), though as Velasco (2008) noted, the idea of recognizing and naming nonsister populations, subspecies, or species as taxa is undesirable if the goal is to generate a taxonomy reflective of genealogical history.

Interestingly, the problem of considering paraphyletic taxa has been recognized by some authors (Lee, 2003; Tobias et al., 2010) and yet interbreeding is prioritized over accurately reflecting evolutionary history. However, if accurately representing evolutionary history and providing names to reflect that history is a primary goal of systematists, then species concepts that group and rank individuals without regard to phylogenetic/genealogical history such as the BSC, Ecological Species Concept (Van Valen, 1976), Cohesion Species Concept (Templeton, 1989), Recognition Species Concept (Paterson, 1985), or Genetic Species Concept (Mallet, 1995) are problematic and poorly communicate that history.

Maintaining paraphyletic species also affects tree inference and downstream application of trees for other avenues of research. Applying the rank of subspecies can prevent accurate study of evolutionary history if terminal tips are composed of grouped nonsister lineages for inferring phylogeny (Ruane et al., 2014). Additionally, many subspecies continue to persist in taxonomies that do not represent lineages, but rather as classification artifacts or handles of conveniences (i.e., legacy subspecies). These legacies are not lineages and therefore placing those on trees will not reflect lineage divergence.

This creates a difficult problem for tree inference and classification above the species when terminals in a phylogeny could be a combination of species as lineages, lineage subspecies, species containing non‐sister subspecies, and legacy subspecies (Yaxley & Foley, 2019). Only the first two categories of terminal units would be useful for inferring phylogeny, and for tree construction, lineage subspecies are equivalent to species. Of course, this affects downstream approaches for inferring gene flow, incomplete lineage sorting, historical demography, and macroevolutionary and macroecological processes such as trait evolution, biogeographic inference, diversification, and community assembly (Smith et al., 2018). No tree‐based inference method gives expectations for how terminal taxa (or OTUs) form, and thus cannot accommodate tree distortion (Velasco, 2008) potentially misleading phylogenetic comparative methods.

## 6 PRAGMATIC ISSUES WITH SUBSPECIES IN ETHICS, POLICY, AND CONSERVATION

Based on our above discussion of the philosophical and empirical issues with the subspecies category, there are also several crucial considerations for biodiversity ethics, policy implications, and conservation management (see Pyron & Mooers, 2022). The intersection between values, conservation, and taxonomy is complex and has received extensive attention in the past (Mace, 2004; Moritz, 1994). Based on our formalization of the philosophical and empirical nature of species and the inapplicability of subspecies, we offer a few additional comments.

Some authors have suggested that subspecies may play a useful role in conservation management through greater applicability of policy and legal protections. For instance, Phillimore and Owens (2006) concluded that “subspecies may, in fact, be of considerable conservation utility, as proxies for the sub‐structure found within species.” Yet, as subspecies cannot be defined coherently as the outcome of evolutionary processes that differ from species, it is just as likely that legal protections and management practices will be misled by a focus on arbitrarily named intraspecific taxa (Zink, 2004). Correspondingly, if “subspecies” are found to represent evolutionary significant units (ESUs) in a phylogenetic context produced by historical evolutionary processes (sensu Crandall et al., 2000), we have argued that this is prima facie evidence that they are, in fact, species. Ranking them as such therefore increases their capacity for legal protection under nearly all policy frameworks worldwide.

If taxonomic rank is derived from the degree of reproductive isolation, and considering the complex nature of hybridization, then with regard to conservation Allendorf et al. (2001) is correct in stating “Any policy that deals with hybrids must be flexible and must recognize that nearly every situation involving hybridization is different enough that general rules are not likely to be effective.” Although not solving the problem of population or species protection, it must be realized that there is an unintended feedback loop when recognizing rank given the variation in what is meant by reproductive isolation over space and time and across the genome with regard to conservation status. On the other hand, extinction via hybridization at least acknowledges species existence as unique evolutionary lineages with reticulation (de Queiroz, 2005; Rhymer & Simberloff, 1996).

Accordingly, if one adopts a historical, phylogenetically based species concept that recognizes species as the fundamental unit and primary product of the evolutionary process (Hull, 1976; Nathan & Cracraft, 2020), this reduces the potential for idiosyncratic mismatches between policy aims and empirical taxonomic conclusions. Generally, no one would argue for taxonomic decisions to be made for the sole purpose of achieving a policy outcome, which would undermine both the legal process and scientific method. Rather, some have suggested that recognition of “subspecies” can promote policy aims of conserving ESUs (e.g., Braby et al., 2012). There are two major problems with this.

The first is that it saddles the science of taxonomy with additional aims and considerations that are outside of its remit. The goal of taxonomy, we reiterate here, is to discover species as the fundamental unit of evolution and infer relationships among those units. If ESUs or subspecies represent evolutionarily distinct, historical phylogenetic units, then they should simply be recognized (and protected) as species. If subspecies do not represent distinct historical evolutionary units, then the rank is being utilized for pragmatic or utilitarian reasons to recognize geographic or morphological (etc.) variants solely for policy and management.

For instance, Frankham et al. (2012) concluded that the Phylogenetic Species Concept (PSC) was incompatible with the aims of conservation biology, because recognizing phylogenetic lineages as species thereby creates legal barriers to the transplant or hybridization of those populations, among other issues. This seems untenable and at best misaligned with, if not outright contradictory to, the empirical aims of taxonomy as a science (Pyron & Mooers, 2022), as the policy implications of a taxonomic decision are subordinate to scientific accuracy. Arguing the reverse, Russello and Amato (2014) concluded that *only* the PSC was sufficiently operationalized to function effectively for conservation and management purposes.

The second, more pragmatic issue is that formal taxonomic recognition is obviously not an intrinsic requirement of legal policy, which can be modified at will, or conservation management, which typically has a specific geographic or population context. As noted by Braby et al. (2012), many major legislative frameworks are not dependent on trinomial nomenclature. Thus, no alteration of empirical taxonomic practice is needed to address any fundamental issue in conservation policy (Haig et al., 2006). Nonhistorical infraspecific units could also make conservation *more* difficult if we want to prioritize species delimitation, but current protections of poorly designated subspecies limit sampling efforts to properly designate species or study the biology of these organisms locally.

If biodiversity has intrinsic value, then the most accurate taxonomy that reflects the real existence and extent of that biodiversity is obviously most desirable for management and policy. The debate over nature's value and biodiversity in particular, addressed in part by the philosophical field of environmental ethics (Brennan & Lo, 2021), is far from settled. There is surprisingly little agreement over basic questions such as whether biodiversity has intrinsic value (as an end unto itself) or only instrumental value (as a means to an end) such as ecosystem services or commercial material (see Maier, 2012; Vellend, 2014). Crucially, is the value of life centered on the individual organism (Agar, 2001) or does it emerge at higher levels, such as the species (Lockwood, 1987)? Would that value extend to infraspecific units such as “subspecies?” Regardless of how one answers these open questions (Callicott, 1989; Norton, 1995), we suggest that subspecies confound these deep issues in conservation and environmental ethics.

Resolving these questions is beyond the scope of the present review. However, we make several basic observations based on our definition of taxonomy as the discovery and classification of natural, concrete species as the fundamental unit and primary outcome of the evolutionary process. If, as Lockwood (1987) and Agar (2001) suggest, value is located in individual organisms, the moral implications derived from species concepts are lessened or alleviated, and the inapplicability of subspecies is primarily limited to the philosophical and empirical issues described earlier. One might question, however, the ethical implication of privileging one set of arbitrarily delineated yet morally equivalent individuals as a subspecies, especially if by doing so they receive differential conservation (Zink, 2004).

Alternatively, perhaps species have intrinsic value (see Sandler, 2012; Smith, 2016). If this is the case, then a logical inference might be that the taxonomy most in accord with the value of biodiversity would be one which recognizes the fundamental units of evolution as species, as we argued for above. Thus, subspecies or other ranks erected based on intrinsic reproductive isolation would distort interpretation of nature's value via the same implied distortions of phylogenetic and evolutionary history outlined by Rosen (1979) and Velasco (2008). This is the mirror‐image conclusion of Frankham et al. (2012).

Finally, perhaps species have only instrumental value, such as for their ecosystem services or their various values to humankind. This would not affect the status of species as ontological individuals produced by the evolutionary process, and thus the instrumental value judgment of species would be orthogonal to the practice of taxonomy as an empirical science. If subspecies are inappropriately confounded with ESUs (see discussion in Braby et al., 2012) as nonhistorical entities erected for purposes related to conservation value (e.g., Frankham et al., 2012), this again burdens taxonomic ranks with nonhistorical secondary considerations which they were not designed to address. As described earlier, the pragmatic aims of such approaches can usually be addressed with nontaxonomic policy and management solutions. Therefore, we argue that in any of these cases, the taxonomic solution most congruent with the value of biodiversity is one which diagnoses and delimits the naturally arising, fundamental units of that biodiversity as an outcome of the evolutionary process, the species.

## 7 TAXONOMIC SOLUTIONS FOR SPECIES FAILING TO SHOW REPRODUCTIVE ISOLATION

Our discussions above are not concerned with species delimitation per se; whether or not subspecies exist is orthogonal to how species are delimited, a question which has many approaches (Carstens et al., 2013). Nevertheless, readers may rightfully ask how this understanding should affect their interpretation of empirical data. Correspondingly, we wish to counteract three potential misreadings of our discussion. First, the decision of whether an independently evolving metapopulation lineage exists as a species may not easily be answered objectively. In nearly all instances, investigators will still have to make a decision with some degree of subjectivity. Here, we reiterate previous authors that such determinations must appeal to empirical data that are derived from an understanding of the evolutionary history of populations with explicit reference to their historical genealogical relationships (Leaché et al., 2019). However, the question still carries a philosophical component. Thus, we do not simply advocate treating population clusters identified within species using methods such as BPP, PHRAPL, or STRUCTURE as species (see Sukumaran & Knowles, 2017); the computational method alone cannot make the decision as to whether the entities delimited as species (by such technique) correspond to actual species.

Second, intraspecific genetic and phenotypic variation is widespread and abundant. This provides the rich texture of evolutionary biology, and population‐level differentiation is one of the primary avenues by which we learn about the evolutionary process. Taking our modestly reductionist view of the ontological nature of species does not in any way compress or limit the study of populations across the phylogeography–phylogenetics continuum (Edwards et al., 2016). Rather, we argue that there is a philosophical limit of the resolution of taxonomy as a science in recognizing the evolutionary individual, the species, as the fundamental unit. Note that we are not (as explained earlier) saying that there is a *threshold* of divergence beyond which incipient lineages become species; this is a continuum in nature. Rather, there is an epistemological point beyond which we cannot meaningfully *detect* this divergence; diagnosable lineages should be delimited as species. Groups below this level cannot be recognized taxonomically, but nonetheless remain potent sources of data for ecology and evolution.

Thus, during species delimitation we are attempting to ascertain detectable infraspecific variation which has accumulated to such a degree as to cross the detectable “species event‐horizon” and merit taxonomic recognition. We argue that it makes no sense to speak of infraspecific groups beyond that boundary; otherwise, we are asking about the taxonomic status of nontaxonomic entities. We have shown above that if such entities are historical and independent, they are simply species, and the boundary in that instance should be adjusted accordingly. If the populations are not historical and independent (e.g., incompletely diverged sublineages or populations diagnosed by nonphylogenetic characteristics), then pasting them on as subordinate units to an evolutionary system of classification is a counterproductive attempt to fuse nonequivalent processes and patterns. However, studying, describing, and understanding such infraspecific genetic and phenotypic variation is still an invaluable pursuit.

Finally, we note that subspecies are a regulated rank in the International Code of Zoological Nomenclature (ICZN, 1999). Beyond advocating for cessation of further descriptions or utilization of subspecies, we are not suggesting any major or substantive alteration of Code‐based taxonomic practice. Just because subspecies names are regulated by the Code does not mean that subspecies are real biological entities or phenomena, or that taxonomists have to use them; it simply provides rules and recommendations for their formation, availability, and validity as nomina in the species series. We contend that subspecies should not be used in active or new taxonomies. Importantly, the existence of subspecies in historical literature provides a rich vein of taxonomic hypotheses to be tested using new genomic datasets and methods, and the Code continues to provide a robust framework for their interpretation in a coherent taxonomy.

## AUTHOR CONTRIBUTIONS

**Frank Burbrink:** Conceptualization (equal); methodology (equal); project administration (lead); writing – original draft (lead); writing – review and editing (supporting). **Brian I. Crother:** Conceptualization (supporting); writing – review and editing (supporting). **Brian Tilston Smith:** Conceptualization (supporting); writing – review and editing (supporting). **Christopher M. Murray:** Conceptualization (equal); writing – review and editing (supporting). **Sara Ruane:** Conceptualization (supporting); writing – review and editing (supporting). **Edward A. Myers:** Conceptualization (supporting); writing – review and editing (supporting). **Robert Alexander Pyron:** Conceptualization (equal); writing – original draft (equal); writing – review and editing (equal).

## CONFLICT OF INTEREST

None declared.

## Data Availability

We do not have any data associated with this manuscript

## References

[ece39069-bib-0001] Abbott, R. J. , & Rieseberg, L. H. (2012). Hybrid speciation. In eLS. John Wiley & Sons, Ltd.

[ece39069-bib-0002] Agar, N. (2001). Life's intrinsic value: Science, ethics, and nature. Columbia University Press.

[ece39069-bib-0003] Allendorf, F. W. , Leary, R. F. , Spruell, P. , & Wenburg, J. K. (2001). The problems with hybrids: Setting conservation guidelines. Trends in Ecology and Evolution, 16, 613–622.

[ece39069-bib-0004] Amadon, D. (1949). The seventy‐five per cent rule for subspecies. Condor, 51, 250–258.

[ece39069-bib-0005] Baack, E. J. , & Rieseberg, L. H. (2007). A genomic view of introgression and hybrid speciation. Current Opinion in Genetics and Development, 17, 513–518. 17933508 10.1016/j.gde.2007.09.001 PMC2173880

[ece39069-bib-0006] Barnard‐Kubow, K. B. , & Galloway, L. F. (2017). Variation in reproductive isolation across a species range. Ecology and Evolution, 7, 9347–9357. 29187973 10.1002/ece3.3400 PMC5696433

[ece39069-bib-0007] Barrowclough, G. F. , Cracraft, J. , Klicka, J. , & Zink, R. M. (2016). How many kinds of birds are there and why does it matter? PLoS ONE, 11(11), e0166307. 27880775 10.1371/journal.pone.0166307 PMC5120813

[ece39069-bib-0008] Barth, J. M. I. , Gubili, C. , Matschiner, M. , Tørresen, O. K. , Watanabe, S. , Egger, B. , Han, Y.‐S. , Feunteun, E. , Sommaruga, R. , Jehle, R. , & Schabetsberger, R. (2020). Stable species boundaries despite ten million years of hybridization in tropical eels. Nature Communications, 11, 1433. 10.1038/s41467-020-15099-x PMC7080837 32188850

[ece39069-bib-0009] Barton, N. H. (1979). The dynamics of hybrid zones. Heredity, 43, 341–359.

[ece39069-bib-0010] Barton, N. H. , & Hewitt, G. M. (1985). Analysis of hybrid zones. Annual Review of Ecology and Systematics, 16, 113–148.

[ece39069-bib-0011] Barton, N. H. , & Gale, K. (1993). Genetic analysis of hybrid zones. In R. G. Harrison (Ed.), Hybrid zones and the evolutionary process (pp. 13–45). Oxford University Press.

[ece39069-bib-0012] Baum, D. A. (1998). Individuality and the existence of species through time. Systematic Biology, 47, 641–653. 12066308 10.1080/106351598260644

[ece39069-bib-0013] Bernier, R. (1984). The species as an individual: Facing essentialism. Systematic Biology, 33, 460–469.

[ece39069-bib-0014] Bock, W. J. (2004). Species: The concept, category and taxon. Journal of Zoological Systematics and Evolutionary Research, 42, 178–190.

[ece39069-bib-0015] Bolnick, D. I. , & Near, T. J. (2005). Tempo of hybrid inviability in centrarchid fishes (Teleostei: Centrarchidae). Evolution, 59, 1754–1767. 16329245

[ece39069-bib-0016] Braby, M. F. , Eastwood, R. , & Murray, N. (2012). The subspecies concept in butterflies: Has its application in taxonomy and conservation biology outlived its usefulness? 106, 699–716.

[ece39069-bib-0017] Brennan, A. , & Lo, Y.‐S. (2021). Environmental ethics. In E. N. Zalta (Ed.), The Stanford encyclopedia of philosophy (fall 2021 edition). Department of Philosophy, Stanford University.

[ece39069-bib-0018] Brogaard, B. (2004). Species as individuals. Biology and Philosophy, 19, 223–242.

[ece39069-bib-0019] Brown, W. L. , & Wilson, E. O. (1954). The case against the trinomen. Systematic Zoology, 3(4), 174–176.

[ece39069-bib-0020] Buggs, R. J. A. (2007). Empirical study of hybrid zone movement. Heredity, 99, 301–312. 17611495 10.1038/sj.hdy.6800997

[ece39069-bib-0021] Burbrink, F. T. , Gehara, M. , McKelvy, A. D. , & Myers, E. A. (2021). Resolving spatial complexities of hybridization in the context of the gray zone of speciation in north American ratsnakes (*Pantherophis obsoletus* complex). Evolution, 75, 260–277. 33346918 10.1111/evo.14141

[ece39069-bib-0022] Burbrink, F. T. , & Ruane, S. (2021). Contemporary philosophy and methods for studying speciation and delimiting species. Ichthyology and Herpetology, 109, 874–894.

[ece39069-bib-0023] Burt, W. H. (1954). Mammalogy: The subspecies category in mammals. Systematic Zoology, 3, 99–104.

[ece39069-bib-0024] Callicott, J. B. (1989). In defense of the land ethic. State University of New York Press.

[ece39069-bib-0025] Carstens, B. C. , Pelletier, T. A. , Reid, N. M. , & Satler, J. D. (2013). How to fail at species delimitation. Molecular Ecology, 22, 4369–4383. 23855767 10.1111/mec.12413

[ece39069-bib-0026] Coleman, K. A. , & Wiley, E. (2001). On species individualism: A defense of the species‐as‐individuals hypothesis. Philosophy in Science, 68, 498–517.

[ece39069-bib-0027] Collins, J. T. (1991). Viewpoint: A new taxonomic arrangement for some north American amphibians and reptiles. Herpetological Review, 22, 42–43.

[ece39069-bib-0029] Coyne, J. A. , & Orr, H. A. (2004). Speciation. Sinnauer Associates Inc.

[ece39069-bib-0030] Cracraft, J. (1983). Species concepts and speciation analysis. In R. F. Johnston (Ed.), Current ornithology (pp. 159–187). Springer US.

[ece39069-bib-0031] Crandall, K. A. , Bininda‐Emonds, O. R. , Mace, G. M. , & Wayne, R. K. (2000). Considering evolutionary processes in conservation biology. Trend in Ecology and Evolution, 15, 290–295. 10.1016/s0169-5347(00)01876-0 10856956

[ece39069-bib-0032] Crifasi, R. R. (2007). A subspecies no more? A mouse, its unstable taxonomy, and western riparian resource conflict. Cultural Geographies, 14, 511–535.

[ece39069-bib-0033] Darwin, C. (1859). On the origin of species by means of natural selection, or the preservation of favoured races in the struggle for life. John Murray. PMC5184128 30164232

[ece39069-bib-0034] de Queiroz, K. (1988). Systematics and the Darwinian revolution. Philosophy of Science, 55, 238–259.

[ece39069-bib-0035] de Queiroz, K. (1997). The Linnaean hierarchy and the evolutionization of taxonomy, with emphasis on the problem of nomenclature. Aliso: A Journal of Systematic and Floristic Botany, 15, 125–144.

[ece39069-bib-0036] de Queiroz, K. (1998). The general lineage concept of species, species criteria, and the process of speciation. In D. J. Howard & S. H. Berlocher (Eds.), Endless forms: Species and speciation (pp. 57–75). Oxford University Press.

[ece39069-bib-0037] de Queiroz, K. (2005). A unified concept of species and its Consequences for the future of taxonomy. Proceedings of the California Academy of Sciences, 56, 196–215.

[ece39069-bib-0038] de Queiroz, K. (2007). Species concepts and species delimitation. Systematic Biology, 56, 879–886. 18027281 10.1080/10635150701701083

[ece39069-bib-0039] de Queiroz, K. (2020). An updated concept of subspecies resolves a dispute about the taxonomy of incompletely separated lineages. Herpetological Review, 51, 459–461.

[ece39069-bib-0190] de Queiroz, K. (2021). Response to criticisms of an updated subspecies concept. Herpetological Review, 52(4), 773–776.

[ece39069-bib-0040] de Queiroz, K. (2022). Response to criticisms of an updated subspecies concept. Herpetological Review, 52(4), 773–776.

[ece39069-bib-0041] Descimon, H. , & Mallet, J. (2009). Bad species. In J. Settele , T. G. Shreeve , M. Konvicka , & H. V. Dyck (Eds.), Ecology of butterflies in Europe (pp. 219–249). Cambridge University Press.

[ece39069-bib-0042] Dufresnes, C. , Brelsford, A. , Jeffries, D. L. , Mazepa, G. , Suchan, T. , Canestrelli, D. , Nicieza, A. , Fumagalli, L. , Dubey, S. , Martínez‐Solano, I. , Litvinchuk, S. N. , Vences, M. , Perrin, N. , & Crochet, P.‐A. (2021). Mass of genes rather than master genes underlie the genomic architecture of amphibian speciation. Proceedings of the National Academy of Sciences of the United States of America, 118, e2103963118. 34465621 10.1073/pnas.2103963118 PMC8433553

[ece39069-bib-0043] Edwards, J. G. (1954). A new approach to infraspecific categories. Systematic Biology, 3, 1–20.

[ece39069-bib-0044] Edwards, S. V. , Potter, S. , Schmitt, C. J. , Bragg, J. G. , & Moritz, C. (2016). Reticulation, divergence, and the phylogeography‐phylogenetics continuum. Proceedings of the National Academy of Sciences of the United States of America, 113, 8025–8032. 27432956 10.1073/pnas.1601066113 PMC4961137

[ece39069-bib-0045] Endler, J. A. (1977). Geographic variation, speciation, and clines. Princeton University Press. 409931

[ece39069-bib-0046] Ereshefsky, M. (1992). The units of evolution: Essays on the nature of species. Journal of the History of Biology, 25, 500–501.

[ece39069-bib-0047] Figueiró, H. V. , Li, G. , Trindade, F. J. , Assis, J. , Pais, F. , Fernandes, G. , Santos, S. H. D. , Hughes, G. M. , Komissarov, A. , Antunes, A. , Trinca, C. S. , Rodrigues, M. R. , Linderoth, T. , Bi, K. , Silveira, L. , Azevedo, F. C. C. , Kantek, D. , Ramalho, E. , Brassaloti, R. A. , … Eizirik, E. (2017). Genome‐wide signatures of complex introgression and adaptive evolution in the big cats. Science Advances, 3, e1700299. 28776029 10.1126/sciadv.1700299 PMC5517113

[ece39069-bib-0048] Frankham, R. , Ballou, J. D. , Dudash, M. R. , Eldridge, M. D. B. , Fenster, C. B. , Lacy, R. C. , Mendelson, J. R. , Porton, I. J. , Ralls, K. , & Ryder, O. A. (2012). Implications of different species concepts for conserving biodiversity. Biological Conservation, 153, 25–31.

[ece39069-bib-0049] Frantz, L. A. F. , Schraiber, J. G. , Madsen, O. , Megens, H.‐J. , Bosse, M. , Paudel, Y. , Semiadi, G. , Meijaard, E. , Li, N. , Crooijmans, R. P. M. A. , Archibald, A. L. , Slatkin, M. , Schook, L. B. , Larson, G. , & Groenen, M. A. M. (2013). Genome sequencing reveals fine scale diversification and reticulation history during speciation in Sus. Genome Biology, 14, R107. 24070215 10.1186/gb-2013-14-9-r107 PMC4053821

[ece39069-bib-0050] Frost, D. R. (2020). Amphibian Species of the World: An Online Reference. Version 6.0 [WWW Document]. Amphibian Species of the World: An Online Reference . http://research.amnh.org/herpetology/amphibia/index.html

[ece39069-bib-0051] Frost, D. R. , & Hillis, D. M. (1990). Species in concept and practice – Herpetological applications. Herpetologica, 46, 87–104.

[ece39069-bib-0052] Frost, D. R. , & Kluge, A. G. (1994). A consideration of epistemology in systematic biology, with special reference to species. Cladistics, 10, 259–294.

[ece39069-bib-0053] Frost, D. R. , Kluge, A. G. , & Hillis, D. M. (1992). Species in contemporary herpetology: Comments on phylogenetic inference and taxonomy. Herpetological Review, 23, 46–54.

[ece39069-bib-0054] Gavrilets, S. (2004). Theories of allopatric and parapatric speciation. In U. Dieckmann , H. Metz , M. Doebeli , & D. Taut (Eds.), The formation of biodiversity through adaptive speciation (pp. 112–139). Oxford University Press.

[ece39069-bib-0055] Ghiselin, M. (1987). Species concepts, individuality, and objectivity. Biology and Philosophy, 2, 127–143.

[ece39069-bib-0056] Ghiselin, M. T. (1974). A radical solution to the species problem. Systematic Biology, 23, 536–544.

[ece39069-bib-0057] Ghiselin, M. T. (1981). Categories, life, and thinking. Behavioral and Brain Science, 4, 269–283.

[ece39069-bib-0058] Ghiselin, M. T. (1997). Metaphysics and the origin of species. SUNY Press.

[ece39069-bib-0059] Gillham, N. W. (1956). Geographic variation and the subspecies concept in butterflies. Systematic Zoology, 5, 110–120.

[ece39069-bib-0060] Gloyd, H. K. , & Conant, R. (1990). Snakes of the Agkistrodon complex: A monographic review. Society for the Study of Amphibians and Reptiles.

[ece39069-bib-0061] Gompert, Z. , Mandeville, E. G. , & Buerkle, C. A. (2017). Analysis of population genomic data from hybrid zones. Annual Review of Ecology, Evolution and Systematics, 48, 207–229.

[ece39069-bib-0062] Gosline, W. A. (1954). Further thoughts on subspecies and trinomials. Systematic Biology, 3, 92–94.

[ece39069-bib-0063] Griffiths, G. C. D. (1974). On the foundations of biological systematics. Acta Biotheoretica, 23, 85–131.

[ece39069-bib-0064] Haig, S. M. , Beever, E. A. , Chambers, S. M. , Draheim, H. M. , Dugger, B. D. , Dunham, S. , Elliott‐Smith, E. , Fontaine, J. B. , Kesler, D. C. , Knaus, B. J. , Lopes, I. F. , Loschl, P. , Mullins, T. D. , & Sheffield, L. M. (2006). Taxonomic considerations in listing subspecies under the U.S. Endangered Species Act. Conservation Biology, 20, 1584–1594. 17181793 10.1111/j.1523-1739.2006.00530.x

[ece39069-bib-0065] Harrison, R. G. , & Larson, E. L. (2014). Hybridization, introgression, and the nature of species boundaries. Journal of Heredity, 105(Suppl 1), 795–809. 25149255 10.1093/jhered/esu033

[ece39069-bib-0066] Hendry, A. P. , Grant, P. R. , Rosemary Grant, B. , Ford, H. A. , Brewer, M. J. , & Podos, J. (2006). Possible human impacts on adaptive radiation: Beak size bimodality in Darwin's finches. Proceedings. Biological Sciences, 273, 1887–1894. 16822748 10.1098/rspb.2006.3534 PMC1797601

[ece39069-bib-0067] Hennig, W. (1966). Phylogenetic systematics. University of Illinois Press.

[ece39069-bib-0068] Hewitt, G. M. (2011). Quaternary phylogeography: The roots of hybrid zones. Genetica, 139, 617–638. 21234647 10.1007/s10709-011-9547-3

[ece39069-bib-0069] Hey, J. (2001). Genes, categories, and species: The evolutionary and cognitive causes of the species problem. Oxford University Press.

[ece39069-bib-0070] Hillis, D. M. (2019). Species delimitation in herpetology. Journal of Herpetology, 53, 3–12.

[ece39069-bib-0071] Hillis, D. M. (2020). The detection and naming of geographic variation within species. Herpetological Review, 51, 52–56.

[ece39069-bib-0191] Hillis, D. M. (2021). “Conceptualizations of species and subspecies: A Reply to the ‘It’s Species All the Way Down View’.” Herpetological Review, 52, 49–50.

[ece39069-bib-0072] Hillis, D. M. , & Wüster, W. (2021). Taxonomy and nomenclature of the *Pantherophis obsoletus* complex. Herpetological Review, 105, 795–809.

[ece39069-bib-0073] Holsinger, K. E. (1984). The nature of biological species. Philosophy in Science, 51, 293–307.

[ece39069-bib-0074] Hull, D. L. (1976). Are species really individuals? Systematic Biology, 25, 174–191.

[ece39069-bib-0075] Hull, D. L. (1990). Science as a process. University of Chicago Press.

[ece39069-bib-0076] Hull, D. L. (1999). On the plurality of species: Questioning the party line. In R. A. Wilson (Ed.), Species: New interdisciplinary essays (pp. 23–48). MIT Press.

[ece39069-bib-0077] Huxley, J. (1938). Clines: An auxiliary taxonomic principle. Nature, 142, 219–220.

[ece39069-bib-0078] ICZN . (1999). International code of zoological nomenclature (4th ed.). International Trust for Zoological Nomenclature.

[ece39069-bib-0079] Inger, R. F. (1961). Problems in the application of the subspecies concept in vertebrate taxonomy. In W. F. Blaird (Ed.), Vertebrate speciation (pp. 262–285). University of Texas Press.

[ece39069-bib-0080] Jackson, N. D. , Carstens, B. C. , Morales, A. E. , & O'Meara, B. C. (2017). Species delimitation with gene flow. Systematic Biology, 66, 799–812. 28003535 10.1093/sysbio/syw117

[ece39069-bib-0082] Kearns, A. M. , Restani, M. , Szabo, I. , Schrøder‐Nielsen, A. , Kim, J. A. , Richardson, H. M. , Marzluff, J. M. , Fleischer, R. C. , Johnsen, A. , & Omland, K. E. (2018). Genomic evidence of speciation reversal in ravens. Nature Communications, 9, 906. 10.1038/s41467-018-03294-w PMC5834606 29500409

[ece39069-bib-0083] Kitcher, P. (1984). Species. Philosophy in Science, 51, 308–333.

[ece39069-bib-0084] Kizirian, D. , & Donnelly, M. A. (2008). The network species model. arXiv [q‐bio.PE], 151, 1–25. 10.48550/arXiv.0808.1590

[ece39069-bib-0085] Köhler, G. (2021). Taxonomy of horned lizards, Genus *Phrynosoma* (Squamata, Phrynosomatidae). Taxonomy, 1(2), 83–115.

[ece39069-bib-0086] Laurin, M. (2008). The splendid isolation of biological nomenclature. Zoologica Scripta, 37, 223–233.

[ece39069-bib-0087] Leaché, A. D. , Zhu, T. , Rannala, B. , & Yang, Z. (2019). The spectre of too many species. Systematic Biology, 68, 168–181. 29982825 10.1093/sysbio/syy051 PMC6292489

[ece39069-bib-0088] Lee, M. S. Y. (2003). Species concepts and species reality: Salvaging a Linnaean rank. Journal of Evolutionary Biology, 16, 179–188. 14635856 10.1046/j.1420-9101.2003.00520.x

[ece39069-bib-0089] Leroy, T. , Louvet, J.‐M. , Lalanne, C. , Le Provost, G. , Labadie, K. , Aury, J.‐M. , Delzon, S. , Plomion, C. , & Kremer, A. (2020). Adaptive introgression as a driver of local adaptation to climate in European white oaks. The New Phytologist, 226, 1171–1182. 31394003 10.1111/nph.16095 PMC7166132

[ece39069-bib-0091] Lockwood, J. A. (1987). The moral standing of insects and the ethics of extinction. Florida Entomologist, 70, 70–89.

[ece39069-bib-0092] Mace, G. M. (2004). The role of taxonomy in species conservation. Philosophical Transaction of the Royal Society B Biological Science, 359, 711–719. 10.1098/rstb.2003.1454 PMC1693347 15253356

[ece39069-bib-0093] Maier, D. S. (2012). What's so good about biodiversity?: A call for better reasoning about nature's value, The International library of environmental, agricultural and food ethics. Springer.

[ece39069-bib-0094] Mallet, J. (1995). A species definition for the modern synthesis. Trends in Ecology and Evolution, 10, 294–299. 21237047 10.1016/0169-5347(95)90031-4

[ece39069-bib-0095] Mallet, J. (2013). Subspecies, semispecies, superspecies. In Encyclopedia of biodiversity (pp. 45–48). Academic Press.

[ece39069-bib-0096] Mallet, J. , Besansky, N. , & Hahn, M. W. (2016). How reticulated are species? BioEssays, 38, 140–149. 26709836 10.1002/bies.201500149 PMC4813508

[ece39069-bib-0097] Mayden, R. L. (2002). On biological species, species concepts and individuation in the natural world. Fish and Fisheries, 3, 171–196.

[ece39069-bib-0098] Mayr, E. (1946). The number of species of birds. The Auk, 63, 64–69.

[ece39069-bib-0099] Mayr, E. (1954). Notes on nomenclature and classification. Systematic Zoology, 3, 86–89.

[ece39069-bib-0100] Mayr, E. (1963). Animal species and evolution. Belknap Press of Harvard University Press.

[ece39069-bib-0101] Mayr, E. (1965). Systematics and the origin of species from the viewpoint of a zoologist. Dover Publications.

[ece39069-bib-0188] Mayr, E. (1969). The biological meaning of species*. Biological Journal of the Linnean Society, 1(3), 311–320. 10.1111/j.1095-8312.1969.tb00123.x

[ece39069-bib-0102] Mayr, E. (1982). Of what use are subspecies? The Auk, 99, 593–595.

[ece39069-bib-0103] McEntee, J. P. , Burleigh, J. G. , & Singhal, S. (2020). Dispersal predicts hybrid zone widths across animal diversity: Implications for species borders under incomplete reproductive isolation. The American Naturalist, 196, 9–28. 10.1086/709109 32552108

[ece39069-bib-0104] Mishler, B. D. , & Brandon, R. N. (1987). Individuality, pluralism, and the phylogenetic species concept. Biology and Philosophy, 2, 397–414.

[ece39069-bib-0105] Mora, C. , Tittensor, D. P. , Adl, S. , Simpson, A. G. B. , & Worm, B. (2011). How many species are there on earth and in the ocean? PLoS Biology, 9, e1001127. 21886479 10.1371/journal.pbio.1001127 PMC3160336

[ece39069-bib-0106] Moritz, C. (1994). Defining “evolutionarily significant units” for conservation. Trends in Ecology & Evolution, 9, 373–375. 21236896 10.1016/0169-5347(94)90057-4

[ece39069-bib-0107] Murray, C. M. , & Crother, B. I. (2016). Entities on a temporal scale. Acta Biotheoretica, 64, 1–10. 26342483 10.1007/s10441-015-9269-5

[ece39069-bib-0108] Nachman, M. W. , & Payseur, B. A. (2012). Recombination rate variation and speciation: Theoretical predictions and empirical results from rabbits and mice. Philosophical Transactions of the Royal Society B: Biological Sciences, 367(1587), 409–421. 10.1098/rstb.2011.0249 PMC3233716 22201170

[ece39069-bib-0109] Nathan, M. J. , & Cracraft, J. (2020). SIX. The nature of species in evolution. In The theory of evolution (pp. 102–122). University of Chicago Press.

[ece39069-bib-0110] Nelson, G. J. , & Platnick, N. I. (1981). Systematics and biogeography: Cladistics and vicariance. Columbia University Press.

[ece39069-bib-0111] Nixon, K. C. , & Wheeler, Q. D. (1990). An amplification of the phylogenetic species concept. Cladistics, 6, 211–223.

[ece39069-bib-0112] Norton, B. G. (1995). Why I am not a nonanthropocentrist: Callicott and the failure of monistic inherentism. Environmental Ethics, 17, 341–358.

[ece39069-bib-0113] Nosil, P. (2008). Speciation with gene flow could be common. Molecular Ecology, 17(9), 2103–2106. 18410295 10.1111/j.1365-294X.2008.03715.x

[ece39069-bib-0114] Nosil, P. (2012). Ecological speciation, Oxford series in ecology and evolution. Oxford University Press.

[ece39069-bib-0115] O'Brien, S. J. , & Mayr, E. (1991). Bureaucratic mischief: Recognizing endangered species and subspecies. Science, 251, 1187–1188. 17799277 10.1126/science.251.4998.1187

[ece39069-bib-0116] O'Hara, R. J. (1993). Systematic generalization, historical fate, and the species problem. Systematic Biology, 42, 231–246.

[ece39069-bib-0117] Padial, J. M. , & De la Riva, I. (2021). A paradigm shift in our view of species drives current trends in biological classification. Biological Reviews, 96, 731–751. 33368983 10.1111/brv.12676

[ece39069-bib-0118] Parkes, K. C. (1982). Subspecific taxonomy: Unfashionable does not mean irrelevant. The Auk, 99, 596–598.

[ece39069-bib-0119] Paterson, H. E. H. (1985). The recognition concept of species. In S. Vrba (Ed.), Species and speciation (pp. 21–29). Transvaal Museum.

[ece39069-bib-0120] Patten, M. A. (2015). Subspecies and the philosophy of science. The Auk, 132, 481–485.

[ece39069-bib-0121] Patten, M. A. , & Unitt, P. (2002). Diagnosability versus mean differences of sage sparrow subspecies. The Auk, 119(1), 35.

[ece39069-bib-0122] Phillimore, A. B. , & Owens, I. P. F. (2006). Are subspecies useful in evolutionary and conservation biology? Proceedings of the Royal Society B, 273, 1049–1053. 16600880 10.1098/rspb.2005.3425 PMC1560251

[ece39069-bib-0123] Pigot, A. L. , & Tobias, J. A. (2015). Dispersal and the transition to sympatry in vertebrates. Proceedings of the Royal Society B: Biological Sciences, 282(1799), 20141929. 10.1098/rspb.2014.1929 PMC4286046 25621326

[ece39069-bib-0124] Prager, E. M. , & Wilson, A. C. (1975). Slow evolutionary loss of the potential for interspecific hybridization in birds: A manifestation of slow regulatory evolution. Proceedings of the National Academy of Sciences of the United States of America, 72, 200–204. 1054495 10.1073/pnas.72.1.200 PMC432270

[ece39069-bib-0125] Price, T. D. , & Bouvier, M. M. (2002). The evolution of F1 postzygotic incompatibilities in birds. Evolution, 56, 2083–2089. 12449494

[ece39069-bib-0126] Pyron, R.A. , Burbrink, F.T. , 2009. Systematics of the common kingsnake Lampropeltis getula Serpentes: Colubridae) and the burden of heritage in taxonomy. Zootaxa 2241, 22–32.

[ece39069-bib-0127] Pyron, R. A. , & Mooers, A. Ø. (2022). The normative postulate problem: Hidden ethics in ecology, evolution, and conservation. Biological Conservation, 270, 109584.

[ece39069-bib-0128] Queiroz, K. (1999). The general lineage concept of species and the defining properties of the species category. In R. A. Wilson (Ed.), Species (pp. 49–89). The MIT Press.

[ece39069-bib-0129] Rand, A. L. , & Traylor, M. A. (1950). The amount of overlap allowable for subspecies. The Auk, 67, 169–183.

[ece39069-bib-0130] Reich, D. (2018). Who we are and how we got here: Ancient DNA and the new science of the human past. Knopf Doubleday Publishing Group.

[ece39069-bib-0131] Remsen, J. V. (2010). Chapter 6: Subspecies as a meaningful taxonomic rank in avian classification. Ornithological Monographs, 67, 62–78.

[ece39069-bib-0132] Rensch, B. (1928). Grenzfälle von Rasse und Art. Journal für Ornithologie, 76, 223–231.

[ece39069-bib-0133] Rensch, B. (1929). Das Prinzip geographischer Rassenkreise und das Problem der Artbildung. Bornträger.

[ece39069-bib-0134] Reydon, T. A. C. (2005). On the nature of the species problem and the four meanings of “species”. Studies in History and Philosophy of Science Part C: Studies in History and Philosophy of Biological and Biomedical Sciences, 36, 135–158. 10.1016/j.shpsc.2004.12.004 16120262

[ece39069-bib-0135] Reydon, T. A. C. (2006). Generalizations and kinds in natural science: The case of species. Studies in History and Philosophy of Science Part C: Studies in History and Philosophy of Biological and Biomedical Sciences, 37, 230–255. 10.1016/j.shpsc.2006.03.003 16769557

[ece39069-bib-0136] Reydon, T. A. C. , & Kunz, W. (2021). Classification below the species level: When are infraspecific groups biologically meaningful? Biological Journal of the Linnean Society, 134, 246–260.

[ece39069-bib-0137] Rhymer, J. M. , & Simberloff, D. (1996). Extinction by hybridization and introgression. Annual Review of Ecology and Systematics, 27, 83–109.

[ece39069-bib-0138] Rieppel, O. (2009). Reydon on species, individuals and kinds: A reply. Cladistics, 26, 341–343. 34875805 10.1111/j.1096-0031.2009.00294.x

[ece39069-bib-0139] Rieppel, O. , & Grande, L. (2007). The anatomy of the fossil varanid lizard *Saniwa ensidens* Leidy, 1870, based on a newly discovered complete skeleton. Journal of Paleontology, 81, 643–665.

[ece39069-bib-0140] Rosen, D. (1979). Fishes from the uplands and intermontane basins of Guatemala: Revisionary studies and comparative geography. Bulletin of the American Museum of Natural History, 162, 5.

[ece39069-bib-0141] Rothschild, L. W. , & Jordan, K. (1895). A revision of the Papilios of the eastern hemisphere, exclusive of Africa. Novitates Zoologicae, 2, 167–463.

[ece39069-bib-0142] Rothschild, L. W. R. B. , & Jordan, K. (1903). A revision of the lepidopterous family Sphingidae. Watson & Viney, Ltd.

[ece39069-bib-0143] Roux, C. , Fraïsse, C. , Romiguier, J. , Anciaux, Y. , Galtier, N. , & Bierne, N. (2016). Shedding light on the grey zone of speciation along a continuum of genomic divergence. PLoS Biology, 14, e2000234. 28027292 10.1371/journal.pbio.2000234 PMC5189939

[ece39069-bib-0144] Ruane, S. , Bryson, R. W., Jr. , Pyron, R. A. , & Burbrink, F. T. (2014). Coalescent species delimitation in milksnakes (genus *Lampropeltis*) and impacts on phylogenetic comparative analyses. Systematic Biology, 63, 231–250. 24335429 10.1093/sysbio/syt099

[ece39069-bib-0145] Rudman, S. M. , & Schluter, D. (2016). Ecological impacts of reverse speciation in threespine stickleback. Current Biology, 26, 490–495. 26804556 10.1016/j.cub.2016.01.004

[ece39069-bib-0146] Russello, M. A. , & Amato, G. (2014). Operationalism matters in conservation: Comments on Frankham et al. (2012). Biological Conservation, 170, 332–333.

[ece39069-bib-0147] Ryan, S. F. , Deines, J. M. , Scriber, J. M. , Pfrender, M. E. , Jones, S. E. , Emrich, S. J. , & Hellmann, J. J. (2018). Climate‐mediated hybrid zone movement revealed with genomics, museum collection, and simulation modeling. Proceedings of the National Academy of Sciences of the United States of America, 115, E2284–E2291. 29463695 10.1073/pnas.1714950115 PMC5877999

[ece39069-bib-0148] Sandler, R. (2012). Intrinsic value, ecology, and conservation. Nature Education Knowledge, 3, 4.

[ece39069-bib-0189] Sangster, G. (2014). The application of species criteria in avian taxonomy and its implications for the debate over species concepts. Biological Reviews, 89(1), 199–214. Portico. 10.1111/brv.12051 23869749

[ece39069-bib-0149] Schmickl, R. , Marburger, S. , Bray, S. , & Yant, L. (2017). Hybrids and horizontal transfer: Introgression allows adaptive allele discovery. Journal of Experimental Botany, 68, 5453–5470. 29096001 10.1093/jxb/erx297

[ece39069-bib-0150] Seehausen, O. (2006). Conservation: Losing biodiversity by reverse speciation. Current Biology, 16(9), R334–R337. 16682344 10.1016/j.cub.2006.03.080

[ece39069-bib-0151] Seehausen, O. , van Alphen, J. J. M. , & Witte, F. (1997). Cichlid fish diversity threatened by eutrophication that curbs sexual selection. Science, 277(5333), 1808–1810.

[ece39069-bib-0152] Servedio, M. R. , & Hermisson, J. (2020). The evolution of partial reproductive isolation as an adaptive optimum. Evolution, 74, 4–14. 31721186 10.1111/evo.13880

[ece39069-bib-0153] Simpson, G. G. (1961). Principles of animal taxonomy. Columbia University Press. 10.1126/science.133.3464.1589 17781120

[ece39069-bib-0154] Singhal, S. , & Moritz, C. (2013). Reproductive isolation between phylogeographic lineages scales with divergence. Proceedings of the Royal Society B, 280, 20132246. 24107536 10.1098/rspb.2013.2246 PMC3813342

[ece39069-bib-0155] Smith, B. T. , Bryson, R. W., Jr. , Mauck, W. M., 3rd , Chaves, J. , Robbins, M. B. , Aleixo, A. , & Klicka, J. (2018). Species delimitation and biogeography of the gnatcatchers and gnatwrens (Aves: Polioptilidae). Molecular Phylogenetics and Evolution, 126, 45–57. 29551521 10.1016/j.ympev.2018.03.012

[ece39069-bib-0156] Smith, H. M. , & White, F. N. (1956). A case for the trinomen. Systematic Zoology, 5, 183.

[ece39069-bib-0157] Smith, I. A. (2016). The intrinsic value of endangered species. Routledge.

[ece39069-bib-0158] Sokal, R. R. , & Crovello, T. J. (1970). The biological species concept: A critical evaluation. The American Naturalist, 104, 127–153.

[ece39069-bib-0159] Starrett, A. (1958). What is the subspecies problem? Systematic Biology, 7, 111–115.

[ece39069-bib-0160] Stelkens, R. B. , Young, K. A. , & Seehausen, O. (2010). The accumulation of reproductive incompatibilities in African cichlid fish. Evolution, 64, 617–633. 19796149 10.1111/j.1558-5646.2009.00849.x

[ece39069-bib-0161] Stresemann, E. (1975). Ornithology from Aristotle to the present. Harvard University Press.

[ece39069-bib-0162] Sukumaran, J. , & Knowles, L. L. (2017). Multispecies coalescent delimits structure, not species. Proceedings of the National Academy of Sciences of the United States of America, 114, 1607–1612. 28137871 10.1073/pnas.1607921114 PMC5320999

[ece39069-bib-0163] Taylor, E. B. , Boughman, J. W. , Groenenboom, M. , Sniatynski, M. , Schluter, D. , & Gow, J. L. (2006). Speciation in reverse: Morphological and genetic evidence of the collapse of a three‐spined stickleback (*Gasterosteus aculeatus*) species pair. Molecular Ecology, 15, 343–355. 16448405 10.1111/j.1365-294X.2005.02794.x

[ece39069-bib-0164] Taylor, S. A. , & Larson, E. L. (2019). Insights from genomes into the evolutionary importance and prevalence of hybridization in nature. Nature Ecology and Evolution, 3, 170–177. 30697003 10.1038/s41559-018-0777-y

[ece39069-bib-0165] Templeton, A. R. (1989). The meaning of species and speciation: Genetic perspective. In D. Otte & J. A. Endler (Eds.), Speciation and its Consequences (pp. 3–27). Sinauer Associates.

[ece39069-bib-0166] Tobias, J. A. , Seddon, N. , Spottiswoode, C. N. , Pilgrim, J. D. , Fishpool, L. D. C. , & Collar, N. J. (2010). Quantitative criteria for species delimitation. Ibis, 152, 724–746.

[ece39069-bib-0167] Torstrom, S. M. , Pangle, K. L. , & Swanson, B. J. (2014). Shedding subspecies: The influence of genetics on reptile subspecies taxonomy. Molecular Phylogenetics and Evolution, 76, 134–143. 24662681 10.1016/j.ympev.2014.03.011

[ece39069-bib-0168] Turbek, S. P. , Browne, M. , Di Giacomo, A. S. , Kopuchian, C. , Hochachka, W. M. , Estalles, C. , Lijtmaer, D. A. , Tubaro, P. L. , Silveira, L. F. , Lovette, I. J. , & Safran, R. J. (2021). Rapid speciation via the evolution of pre‐mating isolation in the Iberá seedeater. Science, 371(6536), eabc0256. 33766854 10.1126/science.abc0256

[ece39069-bib-0169] Uetz, P. , & Stylianou, A. (2018). The original descriptions of reptiles and their subspecies. Zootaxa, 4375, 257–264. 29689772 10.11646/zootaxa.4375.2.5

[ece39069-bib-0170] Uy, J. A. C. , Irwin, D. E. , & Webster, M. S. (2018). Behavioral isolation and incipient speciation in birds. Annual Review of Ecology, Evolution, and Systematics, 49, 1–24.

[ece39069-bib-0171] Van Valen, L. (1976). Ecological species, multispecies, and oaks. Taxon, 25, 233–239.

[ece39069-bib-0172] Velasco, J. D. (2008). Species concepts should not conflict with evolutionary history, but often do. Studies in History and Philosophy of Biological and Biomedical Sciences, 39, 407–414. 19026972 10.1016/j.shpsc.2008.09.007

[ece39069-bib-0173] Vellend, M. (2014). The value of biodiversity: A humbling analysis. Trends in Ecology and Evolution, 29, 138–139.

[ece39069-bib-0174] Vonlanthen, P. , Bittner, D. , Hudson, A. G. , Young, K. A. , Müller, R. , Lundsgaard‐Hansen, B. , Roy, D. , Di Piazza, S. , Largiader, C. R. , & Seehausen, O. (2012). Eutrophication causes speciation reversal in whitefish adaptive radiations. Nature, 482, 357–362. 22337055 10.1038/nature10824

[ece39069-bib-0175] Wang, X. , He, Z. , Shi, S. , & Wu, C.‐I. (2019). Genes and speciation: Is it time to abandon the biological species concept? National Science Review, 7, 1387–1397. 34692166 10.1093/nsr/nwz220 PMC8288927

[ece39069-bib-0176] Wen, D. , Yu, Y. , & Nakhleh, L. (2016). Bayesian inference of reticulate phylogenies under the multispecies network coalescent. PLoS Genetics, 12, e1006006. 27144273 10.1371/journal.pgen.1006006 PMC4856265

[ece39069-bib-0177] Wielstra, B. (2019). Historical hybrid zone movement: More pervasive than appreciated. Journal of Biogeography, 46, 1300–1305.

[ece39069-bib-0178] Wiley, E. O. (1980). Is the evolutionary species fiction?‐a consideration of classes, individuals and historical entities. Systematic Zoology, 29, 76–80.

[ece39069-bib-0179] Wiley, E. O. (1981). Phylogenetics: The theory and practice of phylogenetic systematics. Wiley‐Interscience.

[ece39069-bib-0180] Wilson, E. O. , & Brown, W. L. (1953). The subspecies concept and its taxonomic application. Systematic Zoology, 2, 97.

[ece39069-bib-0181] Yaxley, K. J. , & Foley, R. A. (2019). Reconstructing the ancestral phenotypes of great apes and humans (Homininae) using subspecies‐level phylogenies. Biological Journal of the Linnean Society, 128, 1021–1038.

[ece39069-bib-0182] Zink, R. M. (2004). The role of subspecies in obscuring avian biological diversity and misleading conservation policy. Proceeding of the Royal Society B, 271, 561–564. 10.1098/rspb.2003.2617 PMC1691635 15156912

[ece39069-bib-0183] Zink, R. M. (2014). Homage to Hutchinson, and the role of ecology in lineage divergence and speciation. Journal of Biogeography, 41(5), 999–1006.

[ece39069-bib-0184] Zink, R. M. , & McKitrick, M. C. (1995). The debate over species concepts and its implications for ornithology. The Auk, 112, 701–719.

[ece39069-bib-0185] Ottenburghs, J. (2020). Ghost introgression: Spooky gene flow in the distant past. BioEssays: News and Reviews in Molecular, Cellular and Developmental Biology, 42(6), e2000012. 32227363 10.1002/bies.202000012

[ece39069-bib-0186] Sangster, G. (2009). Increasing numbers of bird species result from taxonomic progress, not taxonomic inflation. Proceedings of the Royal Society B, 276(1670), 3185–3191. 19520805 10.1098/rspb.2009.0582 PMC2817124

[ece39069-bib-0187] Wu, C.‐I. (2001). The genic view of the process of speciation. Journal of Evolutionary Biology, 14(6), 851–865.

